# A synthetic 3D human cerebrovascular model informed by histology for simulating the cortical depth-dependent BOLD fMRI signal

**DOI:** 10.1371/journal.pcbi.1014576

**Published:** 2026-08-03

**Authors:** Mario Gilberto Báez-Yáñez, Jeroen C. W. Siero, Matthias J. P. van Osch, Natalia Petridou

**Affiliations:** 1 Translational Neuroimaging Group, Center for Image Sciences, University Medical Center Utrecht, Utrecht, the Netherlands; 2 Spinoza Centre for Neuroimaging Amsterdam, Amsterdam, the Netherlands; 3 C.J. Gorter MRI Center, Department of Radiology, Leiden University Medical Center, Leiden, the Netherlands; University of Birmingham, UNITED KINGDOM OF GREAT BRITAIN AND NORTHERN IRELAND

## Abstract

Recent advances in functional magnetic resonance imaging (fMRI) using the blood oxygenation level–dependent (BOLD) signal at ultra-high field (≥7T) permit mesoscopic investigations of neurovascular function. However, interpreting the BOLD signal remains challenging because it is an indirect measure of neuronal activity influenced by complex vascular architectures and hemodynamic changes. Existing biophysical models often rely on rodent data, limiting their accuracy for human neuroimaging. We introduce 3D VAMOS (three-dimensional VAscular MOdel based on Statistics), a computational framework that generates synthetic 3D vascular networks for specific human cortical regions. By incorporating histological features, such as vessel volume fractions, tortuosity, and artery-to-vein ratios, 3D VAMOS integrates hemodynamic parameters and biophysical processes to estimate depth-dependent BOLD contributions. To ensure physiological plausibility, we validated 3D VAMOS by comparing simulations of synthetic mouse cortex against realistic models derived from two-photon microscopy. Results showed that regional variability in vessel architecture and cortical thickness significantly modulates laminar BOLD responses. Comparisons between human (visual and motor cortices) and mouse models revealed distinct BOLD profiles reflecting species-specific vascular distributions, where superficial vessels disproportionately influence signal detection. Furthermore, simulations demonstrated that gradient-echo BOLD signals emphasize large-vessel contributions, while spin-echo signals better capture microvascular effects. This highlights a critical sequence-dependent sensitivity in laminar fMRI. Additionally, localized simulations of neuronal activity showed that BOLD profiles depend on vessel-specific changes in blood volume and oxygenation. Thus, 3D VAMOS provides a robust computational framework for understanding BOLD changes across cortical depth for both microvessels and larger intracortical veins. As a computationally efficient and scalable tool, it offers a biologically informed framework to interpret cortical depth-dependent signals, to refine high-resolution imaging protocols, and to explore healthy and pathological brain functions. By bridging the gap between microscopic histology and macroscopic neuroimaging, 3D VAMOS provides a robust foundation for decoding the human brain function.

## Author summary

Interpreting high-resolution functional MRI signals is challenging because the architecture of cortical blood vessels strongly shapes the measured responses. Here, we present 3D VAMOS (three-dimensional VAscular MOdel based on Statistics), a computational framework that generates region-specific anatomically realistic 3D vascular networks of human cortex based on histological features. 3D VAMOS integrates statistical representations of human micro- and macro-vascular architecture with simulations of blood oxygenation and biophysical properties of tissues and simulates cortical depth-dependent BOLD signals. The physiological plausibility of the framework was assessed for the mouse cortex, by comparing simulations based on 3D VAMOS and two-photon microscopy-derived 3D vascular models. Our simulations revealed that vascular architecture alone drive pronounced differences in cortical depth-dependent BOLD profiles across species and cortical regions. Superficial pial vessels dominated the signal, while deeper microvasculature contributed more subtly, emphasizing the importance of pulse sequence-specific sensitivity in interpreting cortical depth-dependent fMRI. 3D VAMOS provides a mechanistic and scalable approach to better understand the relationship between cortical vascular architecture and neural activity for normal and pathological brain function in both human and animal models.

## 1. Introduction

Functional magnetic resonance imaging (fMRI), particularly via the blood oxygenation level-dependent (BOLD) signal, is a cornerstone in human neuroscience, enabling non-invasive mapping of brain activity at increasingly high spatial resolution [1–4]. The BOLD signal arises from complex interactions among cerebral blood flow (CBF), cerebral blood volume (CBV), and cerebral metabolic rate of oxygen (CMRO_2_), in response to neuronal activity. These processes modulate locally the concentrations of deoxygenated hemoglobin ([dHb]) across distinct cerebrovascular compartments, including arteries, capillaries, and veins, thereby shaping the observed BOLD signal [5,6].

With ultra-high field MRI (≥7T), neuroimaging researchers can now achieve submillimeter-resolution imaging of cortical activity in humans and animals, opening new opportunities to investigate brain function at the mesoscopic scale across cortical layers and columns [7–30]. Accurately interpreting cortical depth-dependent BOLD signals remains challenging however because vascular anatomy and intrinsic biophysical processes, such as water diffusion and magnetic susceptibility effects, jointly shape the signal. Understanding thus how vascular architecture, hemodynamic changes, and MRI physics interact is essential for linking BOLD responses to neuronal activity and improving interpretations of brain function [31–33].

Computational modelling has played a pivotal role in this effort. Early models, including Ogawa et al. [2] single-vessel cylinder and subsequent randomly distributed oriented cylinders (RADOC) and microsphere models, incorporated vessel size, blood volume fractions, oxygen saturation (SO_2_), and pulse sequence parameters [34–40]. These models clarify how water diffusion and magnetic susceptibility shape the BOLD signal across magnetic field strengths and pulse sequences [41–43]. However, they lack anatomical realism at the mesoscopic scale, particularly within the laminar and columnar organization of the human cortex.

Subsequent work introduced anatomically realistic 3D vascular models, primarily derived from two-photon microscopy of rodent cortex [44–48]. These models revealed that vascular geometry and orientation relative to the magnetic field strongly influence BOLD signal characteristics [47,49,50]. However, translating rodent-based insights to humans remains challenging because species-specific differences in vascular geometry and topology, density, artery-to-vein ratios, and cortical thickness affect signal generation [51–53]. Directly imaging the human cortical vasculature is highly challenging: two-photon microscopy cannot be applied in humans in-vivo due to its invasiveness; post-mortem reconstruction struggles with dense and deeply embedded microvasculature [54,55], and methods such as Indian ink perfusion or emerging methods such as tissue-clearing with light-sheet microscopy are either invasive, impractical, or restricted to small tissue volumes [56]. Histological reconstruction also requires well-preserved tissue, advanced optical imaging, and substantial computational resources. These limitations emphasize the need for human-specific modelling frameworks that combine anatomical realism with biophysical fidelity.

To meet this need, we introduce 3D VAMOS (three-dimensional VAscular MOdel based on Statistics), a computational framework that generates synthetic, anatomically realistic 3D cortical vascular networks for specific human brain regions. The model constructs microvascular and macrovascular networks using statistical priors from human histological studies, including vessel radius, density across cortical depth, tortuosity, artery-to-vein ratio, and cortical thickness [52,54,55,57–61]. The microvasculature is generated using an enhanced Voronoi tessellation algorithm [50,62], while macrovascular structures are generated using kernel-based stochastic methods.

The 3D VAMOS simulates biophysical mechanisms of BOLD signal formation, including step-wise hemodynamic changes (CBF, CBV, SO_2_), intra- and extra-vascular signal components, and magnetic susceptibility effects and water diffusion in tissue. The framework supports both gradient-echo (GE)- and spin-echo (SE)-BOLD simulations across cortical depths. GE-BOLD, which is most commonly used in fMRI, is highly sensitive to changes in [dHb] but is influenced by macrovascular signals. Although this large-vessel contribution is well-known [16,17,19], it poses a challenge for quantitative interpretation. SE-BOLD, on the other hand, improves microvascular specificity at the expense of lower sensitivity [16,19,34].

By integrating human-specific vascular statistics with mechanistic modelling, 3D VAMOS provides a flexible, physiologically informed platform for simulating cortical depth-dependent BOLD signals, optimizing fMRI pulse sequences, and interpreting neurovascular coupling. This framework advances computational neuroimaging toward more accurate, biologically informed models of human brain function.

## 2. Results

In the human brain, cortical blood vessels are organized into well-defined structures with repetitive topologies [51,56]. These structures consist of a tree-like arrangement of penetrating arteries surrounding a central ascending vein, which collects deoxygenated blood from the microvasculature (arterioles, a mesh-like capillary network, venules) and directs it toward the superficial pial veins [52,54–61,63].

**Fig 1:**
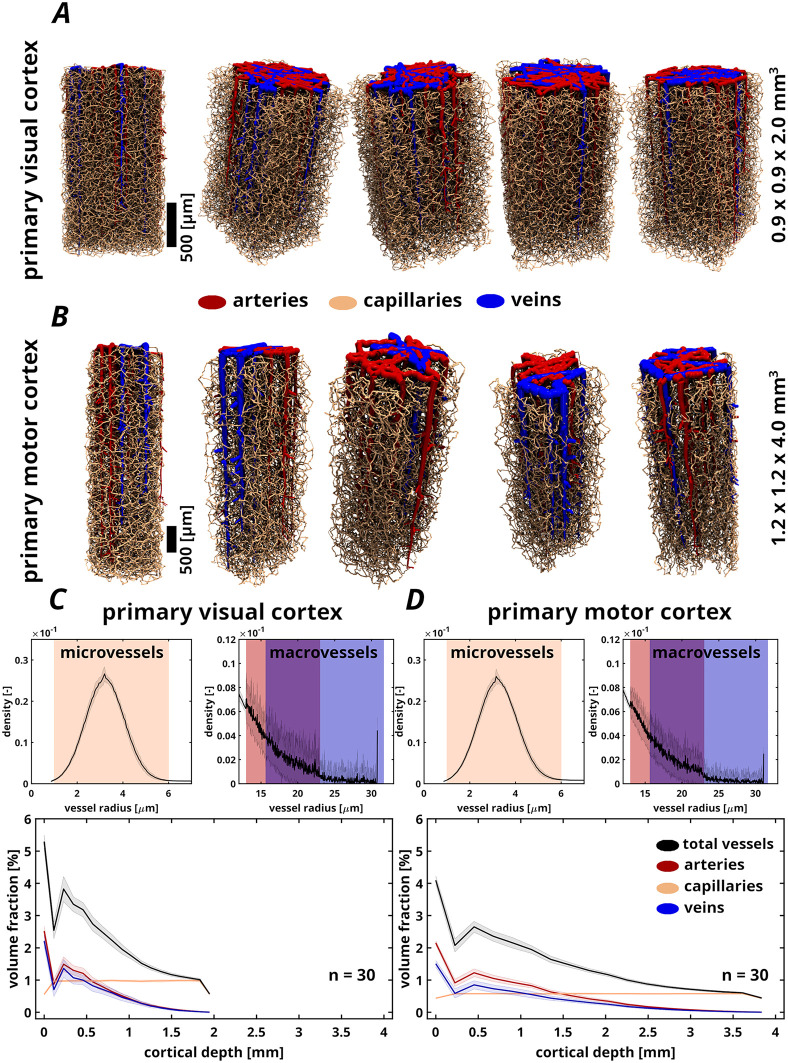
Comparison of representative 3D VAMOS human vascular models: (A) Five representative models of the primary visual cortex (approx. 0.9 x 0.9 x 2.0 mm^3^) and (B) five representative models of the primary motor cortex (approx. 1.2 x 1.2 x 4.0 mm^3^). Vascular features were derived from literature values [57,64]. **(C)** and **(D)** illustrate the distributions of vessel radii (upper graphs) and volume fractions across cortical depth (lower graphs) for both regions. The mean (solid line) and standard deviation (shaded area) of thirty representative models are displayed. In the microvessel distributions, the beige shaded region represents the range of possible values. For macrovessel distributions, red and blue shaded areas represent arterial and venous contributions, respectively; the intersection of these areas indicates the overlap in probability densities between the two vascular compartments.

To demonstrate the capabilities and versatility of the 3D VAMOS computational framework, we generated cortical vascular models emulating the human primary visual and motor cortices (five representative vascular renders per region are shown in Fig 1A and 1B respectively). The models were confined to a simulation space of approximately 0.9 x 0.9 x 2.0 mm^3^ for the primary visual cortex (Fig 1A), and 1.2 x 1.2 x 4.0 mm^3^ for the primary motor cortex (Fig 1B). The 2.0 and 4.0 mm dimension correspond to the reported cortical thickness for these regions [52,61], while the in-plane dimensions were selected arbitrarily. We generated the vascular model rendering using ParaView (open-source software, version 6.0.1).

The models comprised a microvascular compartment (arterioles, capillaries, venules) with vessel radii taken from a Gaussian distribution with mean of 3.235 µm and a standard deviation of 0.850 µm [54,57,65]. Microvessel tortuosity was fixed to 1.2 [65], and microvessel density followed a uniform distribution across cortical depth [52]. The macrovascular compartment (pial and penetrating arteries, pial and ascending veins) was set to an artery-to-vein ratio of ~3:1 [51]. For the primary visual cortex, 42 penetrating arteries with radius in the range of 13 µm to 23 µm, and 14 ascending veins with radius in the range of 15.65 µm to 31.65 µm were modelled. For the primary motor cortex, 85 penetrating arteries and 25 ascending veins were modelled, with vessel radii similar to those in primary visual cortex [57,66]. The number of penetrating arteries and ascending veins was determined based on the simulated in-plane size of each model and the reported spatial distribution values for each cortical region of the brain [51,61]. Macrovessels penetrated to different cortical depths, labelled from A1 to A5 for arteries and V3 to V5 for veins, according to the classification reported in [51]. A minimum radial positioning distance between penetrating arteries and ascending veins of approximately 120 µm was assumed for both modelled cortical regions [60,61].

To demonstrate the reproducibility of generating models for a specific cortical region, Fig 1C and 1D display vessel radius and volume fraction distributions across cortical depth for thirty independently generated vascular models (n = 30; mean (solid line) and standard deviation (shaded area)), for the primary visual and motor cortex, respectively. In both modelled regions, the total volume fraction increased toward the pial surface (located at 0 mm), with arteries exhibiting larger volumes compared to the venous side, while the microvasculature followed a uniform distribution across cortical depth. Similar cortical depth blood volume profiles were observed in both the primary visual and motor cortices, though they differed in cortical thickness and in the steepness of blood volume fraction variation across cortical depth. The total volume fraction at the pial surface in the primary visual cortex (~5%) was slightly higher than in the primary motor cortex (~4%). The microvascular radius distribution followed the imposed Gaussian distribution, while the macrovasculature showed a more uniform radius distribution for arteries and veins.

**Fig 2:**
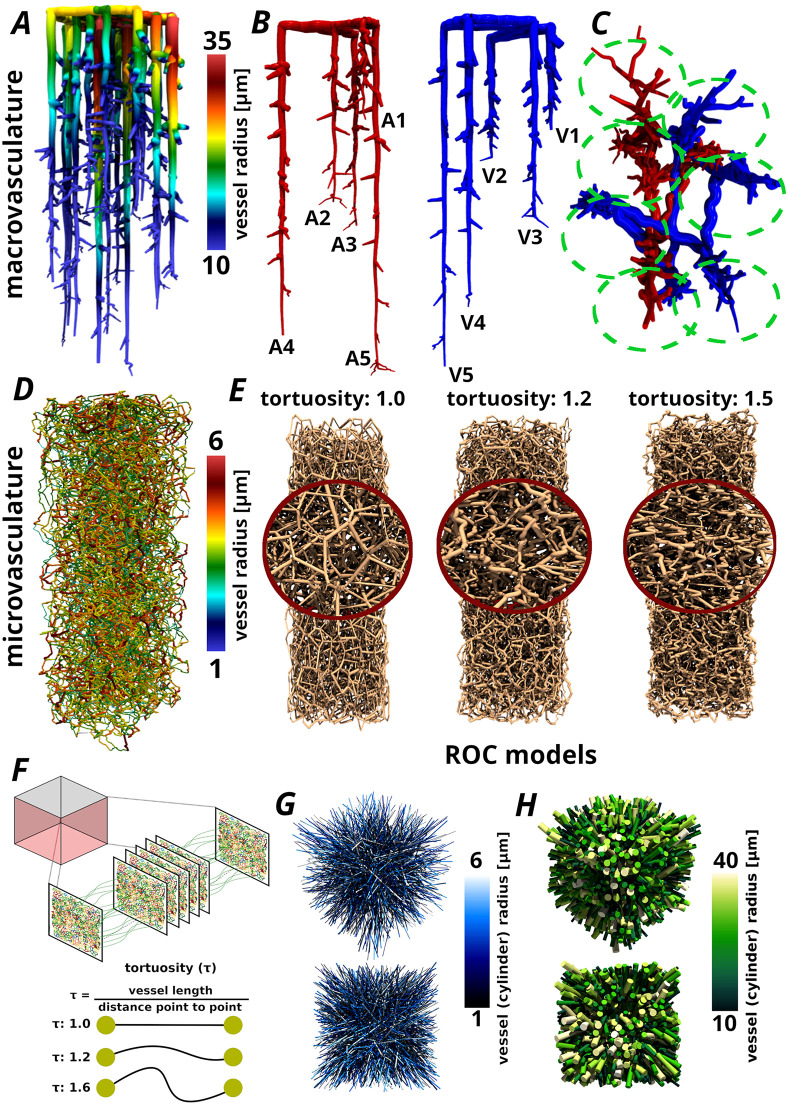
(A) Representative macrovascular architecture showing the vessel radius distribution according to Murray’s law (R^k^parent = R^k^daughter1 + R^k^daughter2 with k = 2) [67,68]. **(B)** Representation of pial and penetrating arteries – from A1 to A5 – and pial and ascending veins – from V1 to V5 – generated with the 3D VAMOS algorithm for different cortical penetration depths as described by histological characteristics [51]. **(C)** Schematic representation of a top-down perspective of spatially confined arterial and venous columnar territories (delineated by green dashed lines), simulating realistic perfusion architecture as observed in-vivo [69]. **(D)** Representative capillary network illustrating the vessel radii spatial distribution ranging from 1 µm to 6 µm. The synthetic capillary bed incorporates a local “smoothing” constraint during network generation to maintain radius continuity across adjacent segments. This ensures biologically plausible morphological properties by preventing unphysiological, abrupt changes in vessel radius at bifurcations or along continuous vessel paths. **(E)** Examples of representative 3D VAMOS microvascular architectures for different levels of vessel tortuosity. The circular images provide a zoomed-in view of each respective model. A vessel tortuosity with a value of 1.0 represents a straight line of edges as computed by the Voronoi tessellation. Tortuosity values larger than 1.0 are simulated using the kernel functions described in Materials and Methods. **(F)** Sketch of the generation of the microvascular structure as described in Materials and Methods. The slabs are tiled using a Voronoi tessellation algorithm and then connected to generate a fully interconnected network. And finally, a sketch of representative randomly distributed oriented cylinders (RADOC) model intended for visually comparing the differences in vascular architecture (microvessels **(G)**; macrovessels **(H)**) between these traditional models and a more realistic one such as the 3D VAMOS model.

Fig 2 presents a detailed illustration of a representative 3D VAMOS cortical vascular model, illustrating the generation of the macrovascular and microvascular architecture including the control of morphological features, such as tortuosity and vessel radius. To quantify the influence of biologically informed vascular architecture on BOLD signal characteristics, we utilized the RADOC model with uniform vessel distributions as a standardized benchmark. This configuration reflects traditional biophysical modelling approaches that historically rely on simplified, homogeneous vascular geometries [2,34]. By maintaining this uniform baseline in RADOC, we were able to systematically isolate and quantify the specific impact of the heterogeneous, laminar-specific vessel distributions incorporated into the 3D VAMOS framework. In Fig 2A, a color-coded 3D rendering of the macrovasculature illustrates how penetrating arteries and ascending veins enter vertically from the pial surface. Vessel radii decreased from a maximum of 35 µm at the surface to 10 µm in deeper cortex, reflecting the branching hierarchy from pial vessels to deeper cortical vessels. The color gradient shows the vessel radius distribution according to Murray’s law (R^k^parent = R^k^daughter1 + R^k^daughter2 with k = 2) [67,68], and demonstrates the structural diversity and caliber of penetrating arteries and ascending veins. Fig 2B illustrates the cortical penetration simulation for five representative penetrating arteries (A1–A5, shown in red) and five ascending veins (V1–V5, shown in blue), extending from the pial surface (A1 and/or V1) to deeper cortical levels (A5 and/or V5), consistent with Duvernoy et al., [51]. These vessel geometries show branching patterns that reproduce a biologically plausible vascular topology, with larger parent branches (R^k^parent) giving rise to smaller daughter branches (R^k^daughter1 and R^k^daughter2) that dive perpendicularly and radially into the cortex. Fig 2C provides a top-down perspective of spatially confined arterial and venous columnar territories (delineated by green dashed lines), simulating realistic perfusion architecture as observed in-vivo [61,63,69]. The microvascular compartment is shown in Fig 2D which forms a dense and spatially homogeneous mesh (1–6 µm radii), critical for oxygen and nutrient delivery into cortical tissue. In Fig 2E, the concept of tortuosity is displayed, comparing microvascular compartments with example tortuosity values τ = 1.0, 1.2, and 1.5, zoomed-in insets illustrate how increasing tortuosity results in more curved vessel paths. Fig 2F displays a sketch of the generation process of the microvascular compartments and the computational implementation of tortuosity, where the vascular volume is divided into multiple sub-regions populated by synthetic microvessels. The schematic illustration depicts tortuosity as the ratio of vessel length to the Euclidean distance between vessel endpoints, with examples showing how a tortuosity of 1.0 corresponds to a straight vessel, while higher values (1.2, 1.5) result in progressively more twisted vessels. For comparison Fig 2G and 2H show RADOC models: a microvascular RADOC model with radii of 1 µm to 6 µm (Fig 2G), and a macrovascular RADOC model with radii of 10 µm to 40 µm (Fig 2H).

**Fig 3:**
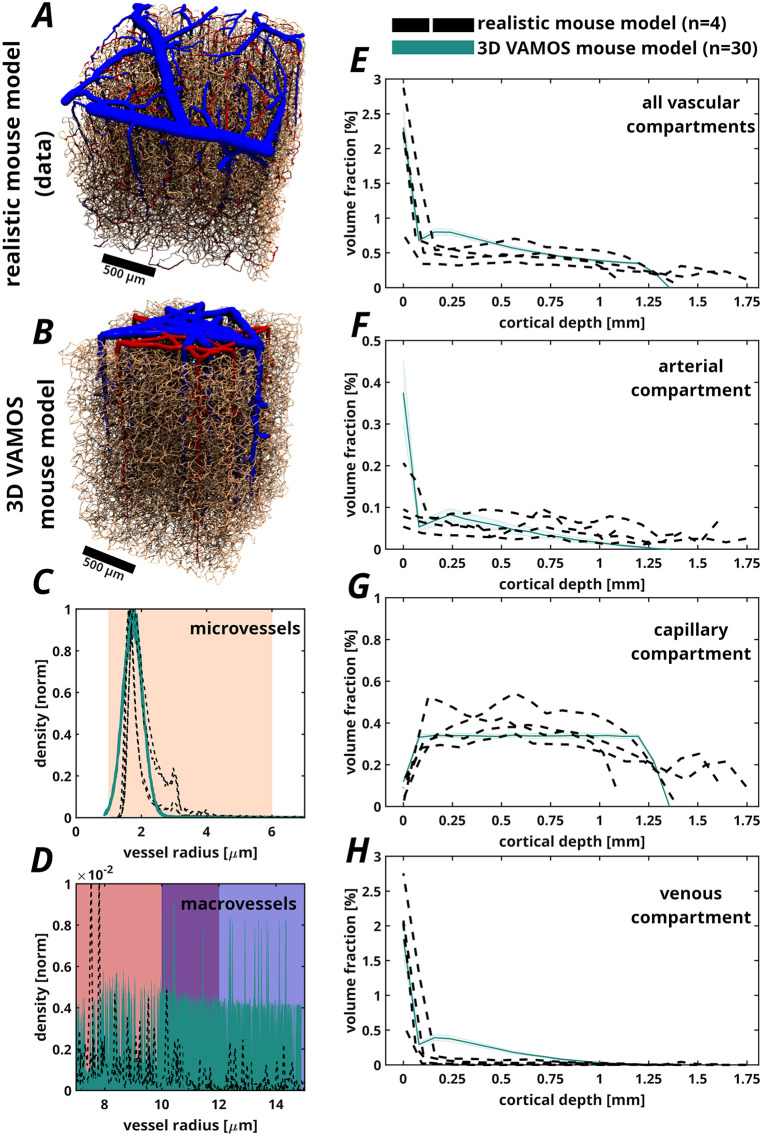
Comparison between realistic and synthetic mouse cortical vasculature. **(A)** 3D rendering of a realistic mouse cortical vascular network reconstructed from imaging data [45], showing macrovasculature (arteries in red, veins in blue) and microvasculature (brown). **(B)** Corresponding representative synthetic vascular network generated using the 3D VAMOS algorithm. **(C)** Normalized vessel radius distribution of the microvascular compartment (vessel radius < 6 μm), for the realistic (black dashed lines, n = 4) and simulated models (green, n = 30). **(D)** Vessel radius distribution of the macrovascular compartment (vessel radius > 6 μm), with color-coded background highlighting arteries (pink and purple), and veins (blue and purple). **(E) – (H)** Volume fraction of vasculature across cortical depth (0–1.75 mm) for all vascular compartments **(E)**, arteries **(F)**, capillaries **(G)**, and veins **(H)**. The mean (solid line) and standard deviation (shaded area) of thirty representative models are displayed; dashed lines (n = 4) indicate individual realistic vasculature models [31,45].

To determine the physiological plausibility of the 3D VAMOS models, we performed a comparative analysis between mouse 3D VAMOS models and realistic 3D vascular models derived from two-photon microscopy of the mouse somatosensory cortex [45]. The vascular features of the synthetic model were derived from literature values [45,49]. Microvessels were modeled with a Gaussian distribution of radii, centered at 1.750 µm with a standard deviation of 0.300 µm. The macrovasculature was generated by imposing five penetrating arteries and fifteen ascending veins (simulating a mouse artery-to-vein ratio of 1:3), with intracortical vessel radii ranging from 7–12 µm for arteries and 10–15 µm for veins (see Materials and Methods). Fig 3 presents a side-by-side comparison, illustrating spatial and quantitative vascular features. Fig 3A and 3B provide a visual comparison. The realistic vascular model (Fig 3A) shows a dense, heterogeneous vascular network with clear distinction of microvasculature, with large ascending veins (blue) and penetrating arteries (red), and a highly interwoven, dense microvascular mesh. The synthetic model (Fig 3B) reproduces a vascular topology qualitatively similar to the biological data, with major vessel trajectories and a comparably distributed capillary network. However, the macrovasculature in the 3D VAMOS model appears somewhat more regular and homogeneous, especially at the pial surface, lacking some of the structural variability observed in the biological counterpart. Fig 3C and 3D compare the vessel radius distributions, distinguishing between microvessels and macrovessels. For microvessels (Fig 3C), both the realistic and simulated models show similar radius distributions, with a prominent peak around ~2 µm. Realistic distributions do not follow an exact Gaussian pattern; instead, they exhibit some skewness compared to the synthetic models, where a Gaussian distribution was imposed. For macrovessels (Fig 3D, radii >6 µm), both models showed two distributions reflecting arteries (pink and purple shaded areas, ~ 6–12 µm), and veins (blue and purple shaded areas, > 10 µm). Fig 3E through Fig 3H present cortical depth-resolved volume fraction distributions for the different vascular compartments, for realistic models (n = 4) and 3D VAMOS models (n = 30, solid line represents mean value and shaded area the standard deviation). For both realistic and 3D VAMOS models the total vascular volume fraction was highest near the cortical surface (0–0.25 mm) and declined sharply with depth, though 3D VAMOS models showed less inter-model variability than the realistic models (Fig 3E). For both models, the arterial compartment showed a smooth decrease in volume fraction from the pial surface to deeper laminae (Fig 3F), while the microvascular volume fraction was distributed more evenly across cortical depth (Fig 3G). The venous compartment showed a very strong surface bias with nearly all venous volume localized in superficial layers (Fig 3H). Across all compartments, 3D VAMOS reproduced cortical depth plots with lower variance across simulation realizations (green lines) compared to the realistic vasculature models (dashed black lines), suggesting that the synthetic vascular model was highly reproducible.

In this study, we aimed to establish a robust framework linking vascular architecture features to BOLD signal formation. To achieve this, we assigned physiologically plausible steady-state hemodynamic parameters, such as blood oxygenation, to each vascular compartment, using established literature values representative of baseline, intermediate, and activated conditions. This approach allowed to systematically isolate the contribution of vascular geometry to the resulting BOLD signal without introducing additional variability from dynamic flow or transient physiological responses [70,71]. By constraining the model in this way, the attributed differences in the simulated signal relate directly to structural features of the vascular network, thereby gaining clearer insight into the role of macro- and micro-vascular organization. Description of the specific parameter choices and their implementation, including vessel-dependent oxygen saturation and size-dependent hematocrit levels, are found in detail in the Materials and Methods sections.

**Fig 4:**
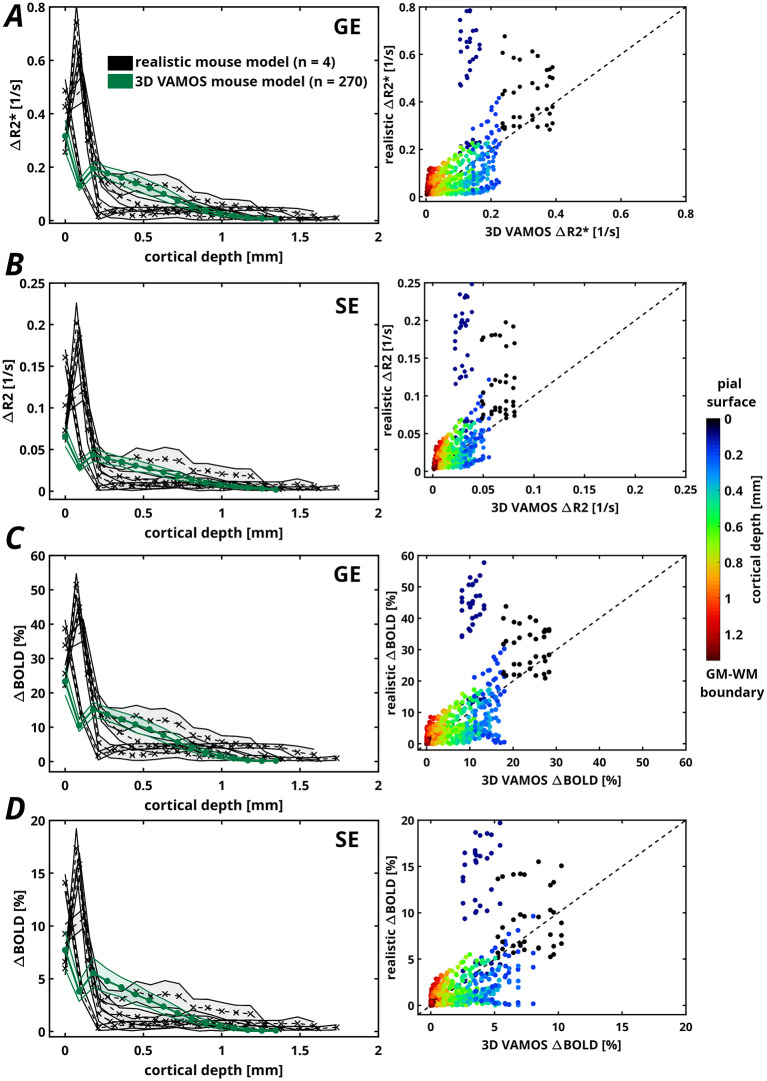
Comparison of cortical depth-dependent relaxation rates and ΔBOLD signal change profiles derived from mouse vascular models based on biological data (n = 4) and 3D VAMOS simulations with matched vascular architectural properties (n = 270). Solid lines with dots represent the mean value computed through the different oxygen saturarion scenarios, while the shaded area represents the standard deviation computed across several Monte Carlo simulations for the synthetic 3D VAMOS mouse models; shaded areas indicate the corresponding standard deviations. Dashed lines with crosses represent the mean values obtained from multiple Monte Carlo simulations for the realistic mouse models; shaded areas indicate the corresponding standard deviations. Panels **(A)** and **(B)** show the cortical depth-dependent ΔR2* and ΔR2 relaxation rates, respectively. Panels **(C)** and **(D)** display ΔBOLD signal changes across cortical depth for GE and SE sequences, respectively. The panels on the right present side-by-side comparisons between relaxation rates and ΔBOLD signal changes, enabling assessment of the relationship between the simulated signals. Each dot represents a comparison between a signal change computed at a specific cortical depth using the 3D VAMOS and the corresponding value obtained from the realistic vascular model.

Hence, we then tested whether 3D VAMOS reproduces physiologically plausible fMRI signal characteristics across cortical depth by comparing the cortical depth-dependent ΔR2*, ΔR2, and ΔBOLD between 3D VAMOS mouse models and realistic mouse vascular models [45] (see Fig 4). ΔR2* and ΔR2 in [1/s] are the changes in transverse relaxation rate caused by [dHb] and reflect the underlying biophysical mechanisms driving the GE-BOLD and SE-BOLD signals, respectively. The BOLD signal changes (ΔBOLD) in [%] computed from the realistic mouse models (n = 4) were plotted individually to allow for better visualization of the specific effects of distinct vascular architectures, while the BOLD signal changes from the 3D VAMOS mouse models (n = 270) were averaged. For both GE and SE, the realistic and synthetic models reproduced the characteristic cortical depth-dependent decay of experimental BOLD fMRI signal changes, with the strongest effects at the pial surface and a progressive decline toward the gray–white matter (GM–WM) border. Signal changes away from the pial surface (>0.25 mm deep) were within the same range for the realistic and synthetic models, indicating that the microvascular networks generated by the 3D VAMOS framework capture the essential statistical properties of the biological vasculature. In the GE simulations (Fig 4A and 4C), the 3D VAMOS models exhibited a qualitatively similar cortical depth-dependent profile to the realistic mouse model, although signal magnitudes were slightly higher in the realistic case within the superficial cortical laminae (<0.25 mm deep). This is also evident in the ΔR2* and ΔBOLD scatter plots, where several points deviated markedly above the identity line. These discrepancies may arise from differences in vascular organization, vessel labelling, and the density of pial and penetrating vessels, which are more structured and homogeneous in the 3D VAMOS-generated networks. In particular, vessel labelling was based on radius-threshold criteria, as described in this manuscript and in [31], which may contribute to differences in vessel classification across vascular architectures. The SE simulations (Fig 4B and 4D) also showed similar agreement between the realistic and 3D VAMOS models, with overall lower amplitudes compared to GE as expected. Both ΔR2 and ΔBOLD SE profiles followed similar trajectories to GE across cortical depth. The scatter plots demonstrate qualitatively similar behavior compared to GE along the identity line, particularly in middle and deeper cortical laminae, albeit with reduced variance. In sum, 3D VAMOS reproduced cortical depth-dependent fMRI signal characteristics consistent with those obtained from realistic models, with some deviations near the pial surface linked to differences in pial and penetrating vessel organization between the models.

**Fig 5:**
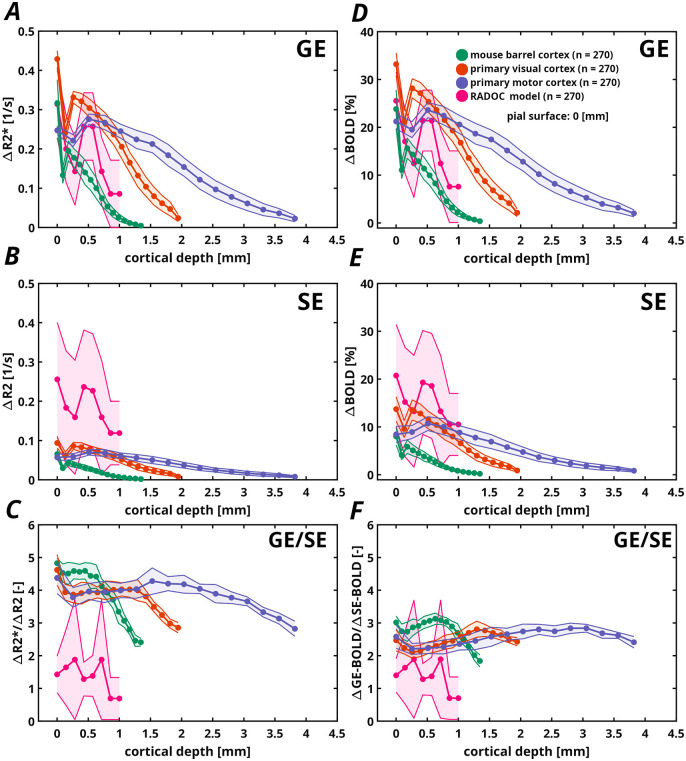
Comparison of relaxation rates, ΔR2* and ΔR2, and BOLD signal profiles across cortical depth, across species (mouse and human) and across cortical regions using distinct simulated vascular architecture characteristics, and the RADOC model. Solid lines with dots represent the mean value computed through the different oxygen saturarion scenarios, while the shaded area represents the standard deviation computed across several Monte Carlo simulations. **(A)** and **(B)** depict the ΔR2* and ΔR2 decay rate across cortical depth, respectively. **(C)** shows the ΔR2*-to-ΔR2 ratio as a surrogate measurement of vessel specificity [16]. **(D)** and **(E)** present the ΔBOLD signal changes across cortical depth for gradient-echo (GE) and spin-echo (SE), respectively; and **(F)** shows the GE-to-SE ΔBOLD ratio as a measurement of vessel specificity [16].

In Fig 5, we present a comparative evaluation of fMRI signal characteristics—ΔR2* and ΔR2 in [1/s], and ΔBOLD signal changes in [%]—across cortical depth for three regions modeled with 3D VAMOS: the mouse barrel cortex (green, n = 270), the human primary visual cortex (orange, n = 270), and the human primary motor cortex (purple, n = 270), with the pial surface marked at 0 mm depth. Moreover, we implemented a cortical depth RADOC model (pink, n = 270) to benchmark our simulations. ΔR2* profiles (Fig 5A) showed a steep decline from the pial surface into deeper laminae across all 3D VAMOS models, but with different amplitudes and penetration depth. The mouse barrel cortex showed the smallest and most superficial ΔR2*, reflecting a limited macrovascular contribution in deeper laminae. The human motor cortex exhibited the most pronounced and sustained ΔR2* throughout the cortical depth, which is consistent with its greater macrovascular presence and a more extensive vascular network that influences MR signal even in deeper laminae. The human visual cortex showed intermediate characteristics, with its ΔR2* peaking sharply near the pial surface and declining more steeply than the motor cortex but less so than the mouse model. ΔR2 profiles (Fig 5B) followed a similar trend for all 3D VAMOS models, peaking near the surface followed by a gradual decay across cortical depth, but with lower amplitudes and more even distribution than ΔR2*. Again, the human motor cortex maintained a more gradual slope and higher baseline, indicating persistent microvascular contributions at greater depths. Fig 5C plots the ratio of ΔR2* to ΔR2, which served as a proxy for the dominance of macrovascular signal contributions relative to microvascular ones [16]. In both human models, ratios were approximately 4 at the surface (<0.25 mm), and decayed to ~3 at deeper cortical depths. The mouse model showed higher superficial ratios, reflecting stronger venous contributions at the pial surface than the human models. ΔBOLD profiles for GE (Fig 5D) closely mirrored the ΔR2*. The motor cortex maintained elevated signal changes for several millimeters reaching 30% near the pial surface in line with the dominance of pial venous vessels in GE BOLD measurements. The mouse model displayed a rapid decay from the surface, and the visual cortex showed intermediate characteristics, both in line with the venous macrovascular volume of these models. SE ΔBOLD (Fig 5E) profiles followed similar trends as GE ΔBOLD but with lower amplitudes and more uniform profiles across cortical depth, especially in deeper laminae. Finally, Fig 5F illustrates the ratio of GE to SE ΔBOLD signal change, highlighting the relative contribution of large vessels to the total BOLD signal [16]. Similarly, as in the relaxation rate ratios, the human models followed similar trends at the superficial laminae (between 2 and 3 [-]), while the mouse model differed greatly, showing an increased bump around 0.5 mm. The RADOC model produced GE BOLD profiles (Fig 5D), qualitatively resembling those of the 3D VAMOS models, suggesting that it captures plausible macrovascular related trends. In SE simulations (Fig 5E), by contrast, the RADOC cortical-depth profiles diverged substantially from 3D VAMOS profiles, suggesting a limited accuracy in capturing microvascular contributions. Moreover, both ratios computed with the RADOC models (Fig 5C and 5F) differed substantially from those obtained with more realistic models.

We next examined how variations in neuronal activity location across cortical depth and associated blood flow changes shape BOLD profiles across cortical depth for GE and SE at 7T, using a volume-flow relationship according to the Davis model [72] with a Grubb’s exponent of 0.25 [73,74]. The simulations were organized into six conditions: three corresponding to different cortical depth locations, and three corresponding to distinct vessel-specific changes in CBV and SO_2_ in response to the simulated neuronal activity.

**Fig 6:**
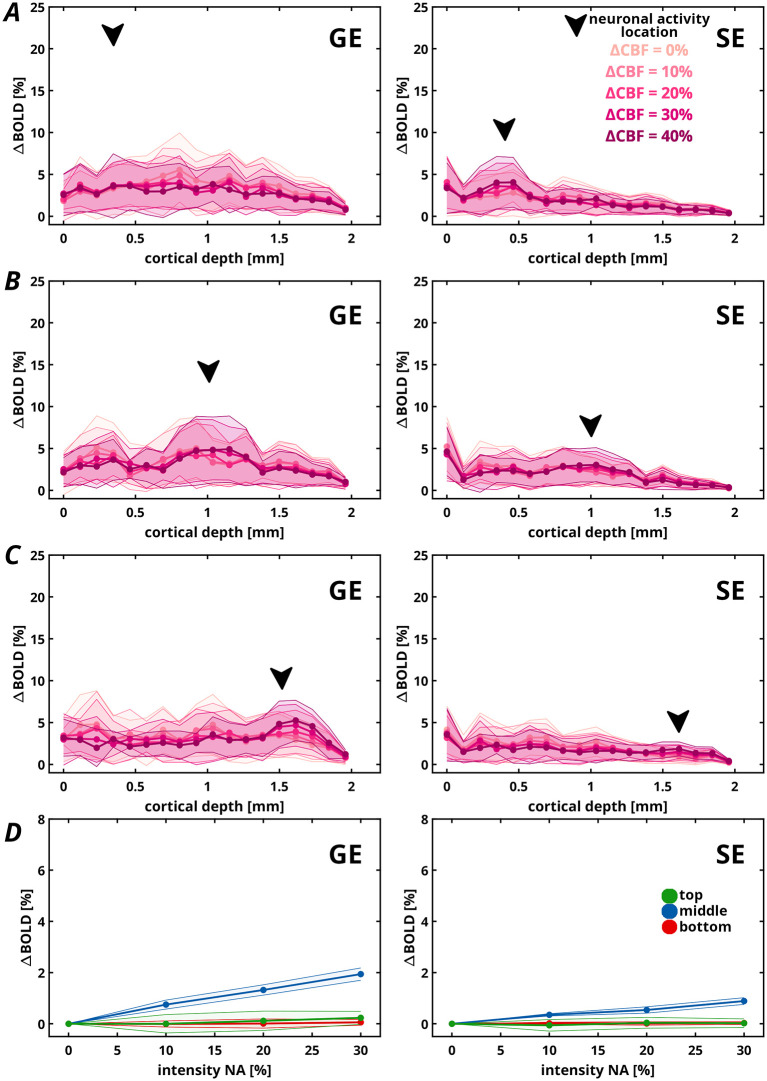
Comparison of cortical depth-dependent ΔBOLD signal profiles across simulated locations of neuronal activity. **(A–C)** Profiles for three simulated activity locations—**(A)** 0.35 mm, **(B)** 1.0 mm, and **(C)** 1.5 mm—assuming local hemodynamic changes where capillaries and arteries dilate (CBV increase of 30%) and SO_2_ increases in veins and capillaries (30%) at the specific activity site. Across all panels, ΔBOLD signal changes are plotted as a function of cortical depth. Multiple traces represent progressively increasing global ΔCBF levels across the entire vascular network, ranging from 0% to 40% (light pink to dark purple). **(D)** Depicts BOLD signal changes across the four simulated proxy effects of neuronal activity intensities (0%, 10%, 20%, and 30%) for the 1.0 mm neuronal activity location. The cortical depth-dependent profiles were grouped into three classes: top (top six sample points), middle (central six sample points), and bottom (deepest six sample points). Each class reflects proxy effects of neuronal activity simulated at distinct cortical depths.

The simulated neuronal activity was centered at superficial (0.35 mm; Fig 6A), middle (1.0 mm; Fig 6B), and deep (1.5 mm; Fig 6C) cortical depths. The vessel-specific changes simulated were as follows: **(1)** Capillaries and arteries dilated locally (i.e., CBV changed by 0–30%), and capillary and venous SO_2_ changed locally (by 0–30%) at the respective activity location. **(2)** Only capillaries dilated locally (CBV change 0–30%), and capillary SO_2_ changed from 0–30% at the respective activity location. **(3)** All vessels dilated locally, with CBV and SO_2_ each changing from 0–30% at the respective activity location. No permutations between CBV and SO_2_ changes were simulated. The results are displayed in Fig 6 for one exemplary condition (assuming local hemodynamic changes where capillaries and arteries dilate (CBV increase of 30%) and SO_2_ increases in veins and capillaries (30%) at the specific activity site) and the remaining simulated conditions are shown in S1 Appendix.

The different simulated neuronal activity locations exhibited distinct BOLD profile amplitudes, depending on the vessel-type–specific hemodynamic changes. Overall, localized BOLD changes at the simulated neuronal activity locations were observable only when local changes in CBV and SO_2_ ocurred in both capillaries and veins. For all conditions, GE showed larger BOLD amplitudes compared to SE. In condition **(1)** (see Fig 6), neuronal activity related peaks were observed in the middle and deeper cortical depth for GE, but was not distinguishable at superficial depth. The middle cortical depth displayed a wider peak compared to the deeper laminae. Conversely, for SE, the peak was more pronounced in the superficial depths, with decreasing amplitude and increasing broadening toward deeper cortical depths. The trade-off among vessel radius, local susceptibility-induced effects, and intrinsic diffusion may explain this behavior, given that SE is highly sensitive to specific combinations of these three BOLD signal drivers, and the simulated conditions lie near the boundary between the narrowing-diffusion regime and the static-dephasing regime [34,36–38]. In condition **(2)** (see *S1 Appendix*), where only capillaries changed their CBV and SO_2_, no clear peak of activity was observed in the BOLD profiles for either sequence. When CBV and SO_2_ was changed in all vessels (condition **(3)**, see *S1 Appendix*), GE BOLD signal changes were pronounced at superficial locations due to the high blood volume in intracortical veins, and diminished in deeper cortical depths. SE showed similar BOLD profiles under condition **(3)**, but with lower amplitudes compared to GE.

## 3. Discussion

### General discussion

Our study addresses a critical gap in understanding how fine-scale vascular architecture shapes high-resolution fMRI signals. We introduce 3D VAMOS, a biologically informed computational framework that generates synthetic 3D models of the human cortical vasculature based on morphological and topological features derived from histological data. By integrating realistic hemodynamics and biophysical processes, 3D VAMOS simulates BOLD signal formation, providing a versatile tool for mesoscale neurovascular modelling and refining ultra-high-field imaging protocols.

The 3D VAMOS algorithm generates fully controlled, well-interconnected vascular networks. It constructs the microvasculature using an aperiodic Voronoi tessellation and kernel functions and builds the macrovasculature solely with kernel functions, both guided by statistical properties previously quantified in the literature. The framework can produce vascular networks of specific human cortical regions, as demonstrated here for the primary visual and motor cortices. Beyond human applications, it also generates vascular architectures for other species, as shown for the mouse barrel cortex. This versatility enables direct comparisons of species- and region-specific vascular organization and its impact on neurovascular coupling and BOLD fMRI signals.

Using these models, we demonstrate that cortical depth-dependent BOLD profiles are strongly shaped by the underlying vascular architecture. Human vascular networks display consistent features that yield comparable depth-dependent BOLD profiles across cortical regions, whereas mouse models diverge markedly from human profiles. These differences emphasize both the importance of accounting for interspecies variability in vascular topology when interpreting cortical depth-dependent fMRI signals and the need for species-specific vascular modelling in neurovascular research.

We also show that the cortical depth of neuronal activity proxies determines the shape of depth-dependent BOLD profiles. Activity near the pial surface produced stronger detectable signals than activity in deeper cortical laminae, reflecting the disproportionate influence of superficial vasculature. Moreover, GE BOLD simulations were more sensitive to neurovascular effects but less specific to the microvasculature than SE BOLD simulations. These findings highlight the importance of considering both vascular depth-dependent contributions and sequence-specific sensitivities when interpreting cortical depth-dependent fMRI signals.

### Comparison of 3D VAMOS mouse model and realistic mouse model

The comparison between the 3D VAMOS mouse model and the realistic vascular model derived from two-photon microscopy data [45] showed that the simulated vascular topology aligns closely with biological data. 3D VAMOS reproduced key features of the realistic vascular architecture, including major vessel trajectories, radius distributions, depth-resolved volume fractions of vascular compartments, and dense microvascular networks. However, some differences emerged. The synthetic macrovasculature in 3D VAMOS appeared more regular and homogeneous than its biological counterpart, particularly at the pial surface. Importantly, vessel labelling in the realistic model directly influences the calculated compartment volume fractions, since vasculature was manually classified using radius thresholds [31]. In addition, the imposed Gaussian distribution in the synthetic microvascular networks produced less skewness than observed in realistic data. While 3D VAMOS exhibited reduced biological heterogeneity, it showed lower inter-model variability than the realistic models, highlighting its reproducibility. These results demonstrate that 3D VAMOS reliably captures key features of cortical vascular architecture and its depth-dependent organization. Although it sacrifices some biological heterogeneity, most notably in pial surface microvasculature, the model provides a consistent, controllable framework for simulating vascular structures, enabling reproducible analyses across multiple scales.

Compared to modelling frameworks developed by Boas and colleagues [75–77], which primarily target oxygen transport and optical signal formation in experimentally imaged microvascular networks, 3D VAMOS offers a distinct advantage: it generates synthetic, anatomically realistic vascular architectures of rodent cortex. Unlike models restricted to small imaged patches, 3D VAMOS scales to larger cortical volumes, integrates hemodynamics and MRI biophysics, and directly simulates mesoscopic neuroimaging signals such as BOLD. This flexibility enables systematic hypothesis testing under diverse anatomical and physiological conditions. Moreover, it is compatible with oxygen transport models, bridging fMRI and optical imaging approaches.

A key benefit of synthetic 3D vascular models lies in overcoming the limitations of microscopy techniques such as two-photon, scanning electron, and light-sheet microscopy [69]. These methods are constrained by finite illumination depth, typically capturing only a few hundred micrometers of cortex. Resulting vascular reconstructions often suffer from deformations, noise, and artifacts, which complicate downstream analyses. Skeletonization—the core step in computational vascular modelling—is highly sensitive to minor boundary perturbations, and even extensive post-processing cannot always distinguish true vascular features from artifacts. Hence, manual vessel classification is often employed using radius thresholds, an approach that introduces some limitations. For example, the apparent lack of pial arteries in the mouse model, can be attributed to the vascular classification methodology employed in the reference dataset [31]. Second, certain venous structures may be misclassified in deeper laminae. These methodological constraints directly affect the estimated compartmental volume fractions. Against this backdrop, the use of such realistic datasets as a benchmark is particularly informative, as it demonstrates the ability of the 3D VAMOS framework to address these gaps by generating vascular networks that are statistically consistent with known histological properties. Thus, the 3D VAMOS framework addresses these issues by rapidly generating fully connected, biologically plausible vascular networks with user-defined parameters at low computational cost. Depending on the required features and volume size, it can generate a complete cortical network within seconds. This capability enables statistical analyses of vascular architectures without relying on a single dataset.

Future improvements in microscopy post-processing will further refine synthetic models like 3D VAMOS. Integrating these advances with machine learning–based segmentation could create a powerful synergy: better experimental data informing more accurate vascular models, which in turn enhance computational tools for analyzing real biological networks.

### On the synthetic human 3D VAMOS model

The main motivation for developing a synthetic 3D vascular model is the limited availability of human cortical vasculature samples [54–56]. Current approaches for 3D visualization of ex-vivo human vascular networks rely on immunohistochemistry labelling combined with microscopy, X-ray microtomography, or optical imaging techniques [78]. However, these methods remain technically challenging, especially for large tissue samples, and tissue degradation and deformation further complicate the acquisition of large volumetric datasets (>1 mm³ isotropic) [51,56,59,79]. The 3D VAMOS framework addresses this limitation by computationally generating human-specific vascular networks based on angioarchitectural characteristics reported in histological and imaging studies [51,59,79].

Traditional vascular models used in BOLD simulations, such as RADOC or microsphere models, fail to structurally capture vascular networks when modelling signal formation at high spatial resolutions [41,42] (see Fig 5). At mesoscopic scales, cortical angioarchitecture follows distinct patterns, such as the microvascular mesh-like network. While RADOC models assume uniform or mixed cylinder sizes within a volume, they cannot represent critical topological features such as penetrating arteries and ascending veins [80]. Particularly, to serve as a benchmark, we chose uniform distributions for the RADOC model for the different laminae as used in traditional biophysical modelling approaches, which historically rely on simplified, uniform vascular geometries. By maintaining this standard configuration for RADOC, we can more clearly isolate and quantify the specific impact that the biologically informed vessel distributions (used in 3D VAMOS) have on BOLD fMRI signal generation. Our results demonstrate that the transition from these simplified uniform assumptions to the more realistic, heterogeneous distributions provided by 3D VAMOS is essential for capturing nuanced laminar BOLD profiles. Moreover, hemodynamic simulations are constrained by the absence of interconnections between cylinders.

The 3D VAMOS model reproduces macro- and microvascular volume fractions of the human cortex [59], as reflected in cortical depth profiles (Figs 1 and 3). This feature is essential for investigating cortical depth specificity in fMRI signals [17,81]. A further advantage is its flexibility: 3D VAMOS can generate vascular networks for different brain regions without being constrained by fixed macrovascular topologies or morphologies. It also enables systematic variation of artery-to-vein ratios to probe how macrovascular organization affects hemodynamics and BOLD signal formation (see Fig 5). Regarding the microvascular architecture, the inclusion of small capillary radii, extending down to approximately 1 µm, represents a modelling choice that warrants careful consideration in light of known limitations in histological measurements. It is well recognized that tissue processing can induce shrinkage, potentially leading to underestimation of in-vivo vessel dimensions. Nevertheless, such measurements provide a consistent and widely used statistical basis for characterizing microvascular architecture, and therefore offer a pragmatic foundation for model parameterization. Importantly, the physiological plausibility of these small vessel sizes is supported by the mechanical properties of red blood cells, which exhibit substantial deformability and are capable of traversing microvessels with diameters smaller than their resting size. This enables effective perfusion even at the smallest scales of the capillary network. These considerations support the inclusion of small capillary radii in the model, as they reflect both the available empirical evidence and the functional characteristics of the microcirculation relevant to BOLD signal formation.

Because the vascular network is fully connected, 3D VAMOS supports local hemodynamic simulations of CBF, CBV, and SO_2_ [50]. Continuous pathways from pial arteries through capillaries to pial veins allow realistic modelling of blood flow dynamics across the full cortical depth [75]. This connectivity makes it possible to impose physiologically meaningful boundary conditions at inlets and outlets, such as blood pressure or flow rates, thereby enabling detailed simulations of cerebral circulation [48,82–85]. Beyond global flow constraints, the framework allows compartment-specific manipulations, such as simulating vasodilation or vasoconstriction in targeted vessels to model vascular pathology, neurovascular coupling surrogates [47], or impaired vascular reactivity in disease [86]. Future studies could extend these applications to investigate mean transit time heterogeneity [87], track contrast-agent passage in dynamic susceptibility contrast MRI for stroke research [88], or explore cerebrovascular physiology under controlled gas challenges [28,29].

The computational efficiency of 3D VAMOS further enhances its utility. Generating microvasculature typically requires only seconds (up to a minute), while macrovasculature is generated within a few hundred milliseconds. By contrast, other algorithms for realistic vascular architectures, such as Hartung et al. [85], can take hours depending on vessel properties. This computational efficiency enables high-throughput simulations and enhances statistical power in BOLD studies, as each iteration generates a new vascular morphology while preserving key topological features, analogous to voxel averaging in fMRI data pipelines (see Figs 4–6).

### On the simulated BOLD fMRI signals

We simulated cortical depth-dependent BOLD signal profiles using 3D VAMOS models of the human primary visual cortex, human primary motor cortex, and the mouse barrel cortex. The simulated profiles resembled those reported in experimental studies of cortical depth-dependent fMRI across these regions [7,8,10–14,16,18,19,21,22,24,29,89]. Simulation results revealed that vascular geometry alone can drive markedly different depth-dependent BOLD profiles. In particular, mouse models diverged substantially from human ones. Because venous compartments dominate BOLD formation, mouse depth-dependent profiles exhibited greater signal decay and stronger surface-weighted responses than human profiles. These interspecies differences primarily stemmed from distinct artery-to-vein ratios.

We also observed cortical thickness–dependent effects. In the thicker human motor cortex (~4 mm), relative BOLD signal changes were lower than in the thinner visual cortex (~2 mm), even at identical simulated imaging resolutions (see Fig 5). By contrast, the mouse somatosensory cortex (~1 mm) produced distinct profiles compared to human vascular models, again reflecting species-specific artery-to-vein ratios. While normalization can help compare cortical depth profiles across species and regions, such approaches must explicitly account for underlying vascular differences. These results highlight the importance of considering both cortical thickness and vascular organization when interpreting laminar BOLD data.

Across all models, GE relaxation rates (ΔR2*) increased toward the pial surface, while SE relaxation rates (ΔR2) rose more moderately because magnetic field inhomogeneities are refocused. Despite this refocusing, macrovascular contributions at the cortical surface remained strong, particularly at higher oxygenation levels. In deeper laminae, GE and SE BOLD signals were dominated by tissue R2*. These results suggest that deep gray matter architecture exerts limited influence on depth-dependent BOLD decay, whereas superficial cortical depths are highly sensitive to variations in large-vessel topology. This asymmetry underscores the need for filtering and normalization techniques to reduce pial vessel biases in laminar fMRI analyses [13,14,90].

We further simulated localized neuronal activation by selectively modulating SO_2_ and CBV within defined cortical depths and for vessel type (see Fig 6 and S1 Appendix). This approach isolated the effects of local vascular changes (CBF, CBV, and SO_2_) on the BOLD signal. The simulation results demonstrate that vessel-type–specific hemodynamic responses and the spatial distribution of neuronal activity critically shape the cortical depth-dependent BOLD profiles. In condition (1), where vessels changed their CBV and SO_2_ according to their type, the resulting profiles most closely resembled those reported in experimental fMRI data. This suggests that realistic interaction (coordination) between arterial, capillary, and venous compartments is essential to reproduce the characteristic depth-dependent BOLD signatures. The observed differences between GE and SE profiles reflect their known sensitivity to vascular scale: GE, dominated by larger vessels, produced higher amplitudes and more pronounced responses in superficial cortical depths, whereas SE, which emphasizes microvascular contributions, showed stronger and more localized responses at middle cortical depths. Conversely, condition (2), where only capillaries were modulated, resulted in relatively flat profiles for both sequences, suggesting that isolated capillary dynamics are insufficient to drive the laminar BOLD contrast typically observed in vivo. In condition (3), where all vessels changed their CBV and SO_2_, the markedly larger amplitudes, particularly at superficial depths, highlight the dominant influence of large-vessel dilation on measurable GE signals. These findings emphasize the importance of modelling vascular heterogeneity and depth-dependent reactivity to interpret laminar fMRI data accurately [15,16,26]. The differential sensitivity of GE and SE sequences highlights the need to consider acquisition-specific biases when linking BOLD contrast to underlying neuronal processes. GE sequences, while more sensitive to signal changes, are influenced by draining veins and thus may overrepresent activity in superficial layers. SE sequences, by contrast, better isolate microvascular effects but with reduced amplitude, making them more suitable for mapping activity at the level of cortical microcircuits [9,17,73,74].

To date, five computational frameworks share the fundamental goal of bridging mesoscopic vascular anatomy and hemodynamic responses to predict empirical fMRI changes, whether at the voxel level or across cortical depth [31,41,42,48,80]. These pipelines incorporate varying MRI sequences (e.g., GE, SE, bSSFP), dimensionalities (2D vs. 3D), and field perturbation mechanics (analytical or Fourier-based magnetic field calculations). A shared objective among the laminar frameworks is accounting for how signal contributions across cortical laminae are modulated by large intracortical veins [31,80]. While 3D VAMOS shares this core objective with established tools like Markuerkiaga et al. [80] and Hartung et al. [48], it diverges significantly in dimensionality, species origin, and pipeline customization. While earlier iterations heavily leveraged idealized or static vascular geometries, 3D VAMOS integrates fully customizable, anatomically realistic microvascular networks with dynamic, depth-dependent adjustments [80]. Furthermore, in contrast to our previous work [31], 3D VAMOS introduces the simulation of synthetic, realistic human vascular models, similar to the work of Hartung et al. [48]. This structural advancement highlights the distinct uniqueness of our framework. A current limitation of the 3D VAMOS model, in comparison to the framework by Hartung et al. [48], is the lack of time-dependent hemodynamics across the vascular network. Nevertheless, 3D VAMOS remains advantageous because it allows users to systematically isolate and manipulate intricate spatial parameters, including local artery-to-vein ratios, vessel densities, vessel-dependent oxygen saturation, and tortuosity, within a highly accessible and flexible simulation pipeline.

### Computational demands and simulation runtimes

The simulation runtimes were benchmarked using a standard desktop workstation configuration. The hardware environment utilized a 22-core (Intel Xeon CPU) computer paired with 62 GB of memory RAM. Under these baseline computational conditions, executing a single, complete depth-dependent BOLD simulation experiment for a single brain region required an average runtime of approximately 6 hours. This processing time scales with the structural complexity and spatial dimensions of the target 3D human vascular network, demonstrating that detailed laminar BOLD modelling can be achieved on standard local infrastructure. Nonetheless, the framework is highly scalable; leveraging high-performance computing clusters can drastically reduce overall simulation times and efficiently facilitate the parallel processing required for the statistical averaging of multiple simulation runs.

### Methodological considerations

While the vascular architecture in-vivo is inherently finite, tortuous, and characterized by complex branching networks, the use of the infinite cylinder approximation remains a well-justified simplification within the context of BOLD signal modelling. At the spatial scales relevant for diffusion-mediated signal formation, vessel segments can be considered locally straight relative to the diffusion length of spins, such that the magnetic field perturbations are accurately captured by the infinite cylinder solution. Importantly, the BOLD signal reflects mesoscopic field variations that are effectively averaged over stochastic spin trajectories, thereby reducing sensitivity to localized geometric features such as vessel endpoints or bifurcations. Configurations in which spins reside near vessel end-points or along the longitudinal axis of finite segments constitute only a small fraction of the sampled space and contribute minimally to the overall signal, particularly within a Monte Carlo framework, as used in this work, where spatial averaging and random sampling further mitigate potential biases. Moreover, the inherent heterogeneity of the vascular network acts to suppress systematic errors arising from localized deviations from the idealized geometry. The validity of this approximation has been supported by multiple prior studies, including our own previous work [31,49,50,91], which demonstrate consistency between model predictions, theoretical expectations, and experimental observations [10,47,92]. These considerations suggest that, despite its geometric simplifications, the infinite cylinder model captures the dominant physical mechanisms underlying BOLD contrast and remains appropriate for the scale of the present study.

The current implementation of the 3D VAMOS framework adopts a step-wise approximation of SO_2_ across arterial, capillary, and venous compartments, rather than modelling a continuous physiological gradient along the vascular tree. While oxygen extraction in-vivo occurs progressively, this compartmentalized representation remains a well-established and widely accepted approach in biophysical BOLD modelling [2,47]. The validity of this approximation is supported by the fact that the BOLD signal is predominantly governed by the paramagnetic effects of deoxygenated hemoglobin, which are most pronounced within the capillary and venous compartments. By assigning discrete yet physiologically plausible SO_2_ values to these vascular segments, the model enables isolation of the influence of vascular architecture on laminar BOLD signal formation without introducing the additional complexity of dynamic oxygen transport processes. Moreover, this approach maintains consistency with prior modelling studies and experimental observations derived from compartment-specific pO_2_ measurements [63,69]. Although the step-wise approximation may modestly underestimate intra-capillary heterogeneity in oxygenation, it provides a robust and validated baseline for capturing the dominant spatial characteristics of the BOLD response across cortical depth.

A further limitation of the current framework lies in the assumption of impermeable vessel walls, whereby exchange between intra- and extravascular compartments is not explicitly modelled. While this simplification is appropriate for studies focused on BOLD signal formation, where susceptibility effects dominate, it may become restrictive in the context of more comprehensive physiological modelling, particularly when incorporating cerebral blood flow (CBF) or detailed oxygen transport mechanisms. In such settings, transvascular exchange of water and dissolved oxygen can contribute to the observed signal and should be accounted for explicitly. To address this limitation, ongoing work is focused on extending the framework to include numerical modelling of exchange processes between compartments, thereby relaxing the impermeability assumption [93,94]. This development will enable a more physiologically complete representation of tissue–blood interactions and improve the applicability of the model to a broader range of hemodynamic and metabolic studies.

While the current 3D VAMOS framework focuses on the biophysical mechanisms underlying BOLD signal formation, it does not yet incorporate spatial encoding effects, such as T2* dephasing during readout. As a result, the simulated laminar profiles remain idealized and do not capture the spatial blurring (i.e., point-spread function, PSF) and signal decay characteristics present in experimental fMRI data. Recent studies have shown that acquisition parameters, including echo-train length (ETL) and k-space sampling in EPI readouts, can substantially shift and blur laminar signals, particularly in SE-EPI acquisitions [91]. Therefore, incorporating full spatial encoding into the 3D VAMOS framework represents an important direction for future development, enabling more direct comparisons with experimental 7T data and improving the assessment of the effective spatial specificity and fidelity of different imaging approaches.

Regarding angioarchitecture, although pial arteries form anastomoses to support regions of high perfusion or collateral flow [64], the current model includes only primary pial vessel segments from large feeding arteries. Pial veins, by contrast, do not exhibit anastomoses at any cortical depth [51]. Future algorithm enhancements will incorporate these vascular features to better capture cortical perfusion dynamics.

### Future studies and computational improvements

A key characteristic of the 3D VAMOS framework is its reliance on a comprehensive set of empirically defined vascular and hemodynamic parameters to simulate depth-dependent percent signal changes. While this multi-parameter architecture allows for highly detailed modelling, a rigorous sensitivity analysis evaluating the isolated and joint impact of variables such as the artery-to-vein ratio, vessel radius, baseline CBV, and Grubb’s constant remains a critical next step to fully map the model’s behavior. Given the high dimensionality of this parameter space, a brute-force sensitivity analysis represents a massive computational undertaking that falls outside the foundational scope of the current validation study. To address this, ongoing work is leveraging a biophysics-informed machine learning approach to systematically map the entire parameter space and learn how complex parameter combinations converge to resemble empirical BOLD responses. Furthermore, the utility of the framework will be extended to explore alternative contrast mechanisms, such as vascular space occupancy (VASO) fMRI signals to characterize the sensitivity and specificity of CBV and SO2 alterations. Exploring these interconnected parameters will be vital for refining the predictive accuracy of future laminar fMRI interpretations.

Current functional neuroimaging methods primarily detect hemodynamic (BOLD) signal changes in response to neuronal activation. Our work addresses a fundamental challenge in high-resolution functional neuroimaging: understanding how fine-scale vascular architecture shapes the BOLD fMRI signal. While directly relevant to interpreting cortical depth-dependent fMRI, our computational framework also opens new avenues for investigating cerebrovascular dysfunction in clinical contexts. For example, cerebral small vessel disease (cSVD) [95]—a leading cause of stroke and dementia—is characterized by microvascular damage that disrupts perfusion and oxygen extraction, yet remains poorly understood at the biophysical level [96]. Using 3D VAMOS, we can explore how such vascular alterations influence the hemodynamic response, providing mechanistic insight into observed pathological changes.

Planned developments of the 3D VAMOS framework aim to expand its scope and enhance its physiological realism. Future iterations will simulate additional cortical regions under varied experimental conditions, systematically comparing outcomes to high-quality experimental data for improved validation. As more high-resolution histological and vascular datasets become available, we will refine anatomical assumptions to enhance model accuracy. A key next step involves scaling the model to larger cortical volumes, incorporating the macro-scale geometry of sulci and gyri to capture their specific effects on microvascular architecture and hemodynamics. Furthermore, we plan to broaden time-dependent hemodynamic changes and fMRI simulation capabilities to include diffusion-sensitive regimes, addressing current assumptions of isotropic diffusion across cortical depth [91].

An important avenue for future development lies in extending the framework to incorporate additional imaging contrasts such as VASO and arterial spin labeling (ASL), which are often associated with improved spatial specificity relative to conventional BOLD imaging. In the current implementation, activation-induced changes in physiological parameters, including cerebral blood flow and volume, are introduced as prescribed inputs rather than dynamically simulated, allowing the model to focus on the relationship between vascular architecture and signal formation. Building on this foundation, future work will explicitly model the primary contrast mechanisms underlying VASO and ASL, namely blood volume–dependent signal changes and the delivery and exchange of magnetically labelled arterial blood, respectively. Importantly, such extensions will also account for secondary contributions, particularly BOLD-related signal contamination, which can significantly influence both modalities and interact with the underlying vascular geometry.

## 4. Materials and methods

### 4.1 Generation of a synthetic human 3D VAscular MOdel based on Statistics – 3D VAMOS

A synthetic vascular model is generated using custom MATLAB code (MathWorks, v.2024a). The statistical properties of human cortical vasculature are derived from literature that estimates these properties through histological analysis [51,52,54,55,57]. First, the microvasculature (which in this manuscript includes arterioles, capillaries, and venules) and the macrovasculature (comprising pial arteries and veins, penetrating arteries, and ascending veins) are generated separately. The vascular networks are then integrated by connecting the macrovascular endpoints of both the arterial and the venous compartments to the microvasculature through their nearest proximal vessel in space (i.e., the nearest vessel neighbor), thereby forming a complete and fully connected 3D VAMOS structure. The generation process for each vascular compartment is described in the following sections.

#### Generation of the microvascular architecture based on Voronoi tessellation and kernel functions.

To account for regional variability in cortical thickness and vascular volume fraction across the human cortical gray matter, we considered that different cortical areas exhibit different cortical thicknesses and cerebrovascular volume fractions [97]. For instance, the human primary visual cortex exhibits a cortical thickness of approximately 2 mm [64,66], while the human primary motor cortex is approximately 4 mm thick [98]. Therefore, the initial parameters of the algorithm included the definition of a customized three-dimensional space with specified x, y, and z dimensions (in millimeters), along with a designated vascular volume fraction. The x and y dimensions represent the in-plane parallel to the cortical surface, and the z dimension corresponds to cortical thickness. Within this volumetric space, we assumed that the cortical vasculature extends from the superficial/pial vessels to the cortical gray-white matter (GM-WM) border.

The 3D VAMOS allows for the definition of any desired volumetric vascular dimensions, ranging from hundreds of micrometers to millimeters. This versatility enables modelling not only human vascular networks but also rodent or other species by adjusting vascular properties accordingly (see Figs 1 and 3).

The microvasculature was generated using Voronoi tessellation, resulting in a mesh-like topological network [54,65]. Voronoi tessellations have been shown to effectively represent the capillary bed [99,100].

The simulated volumetric space was divided into a number S of equidistant slabs in the xy-plane. S was calculated based on the desired vascular volume fraction and vessel radii. An aperiodic Voronoi tessellation was generated by fragmenting each of the slabs into tiles that encompass a given set of seed points [62]. The seed points can be distributed within the slab according to any specified probability distribution to simulate different capillary densities across cortical depth [52]. For example, a Gaussian distribution can be simulated in the xz-plane to create larger capillary densities at middle cortical depths, i.e., a larger density of Voronoi tiles in the middle part of the slab. This would reflect histological observations that vascular density can be slightly higher in the middle layers compared to the superficial and granular layers. [52]. Simpler distributions, such as uniform seed distributions, can also be implemented depending on the modelling needs.

Each slab, then, was tessellated using the linear inequalities formed by perpendicular bisectors between any two connected points in the Delaunay triangulation, employing an adapted version of the polytope-bounded aperiodic Voronoi diagram algorithm [62]. Once all the slabs contained tessellations, vessel joints in the i-th slab were connected with their nearest neighbor vessel joint in the (i + 1)-th slab using the shortest Euclidean path. This results in a fully interconnected network structure consisting of vertices, i.e., microvessel joints, and lines connecting those vertices, i.e., microvessel strands.

To further enhance the realism of the microvascular networks, the vertices (microvessel joints) generated through tessellation were displaced orthogonally to the slab by a small distance, typically on the order of tens of micrometers. This displacement introduced a volumetric distribution to the components of each slab, more closely mimicking the three-dimensional structure of real capillary networks.

To generate a closer resemblance to actual capillary beds, an important characteristic to include is the tortuosity of the microvessels [82–84]. The tortuosity (τ) was defined as the ratio of the vessel length between two vessel joints (vertices), to the Euclidean distance between joints (see Fig 2),


$$\begin{array}{c} \tau =\frac{{\text{vessel length}}_{\text{vessel joints}}}{{\text{Euclidean distance}}_{\text{vessel joints}}\;} \end{array}$$ (1)

To generate vessel morphologies consistent with a desired tortuosity, we developed an iterative curve generator that produces anatomically plausible shapes from predefined mathematical functions. Specifically, sinusoidal functions (α-functions; e.g., A sin(ωr + δ), with A, ω, δ randomly sampled) were combined with weighting functions such as Gaussian, gamma, or double-gamma distributions (β-functions; e.g., N(ζ, η), with ζ, η randomly sampled). The product of these α- and β-functions defines a *kernel function*, which governs the curve generation process and ensures that the resulting morphologies satisfy the imposed tortuosity constraints. Representative examples of microvascular tortuosity generated using this approach are shown in Fig 2.

In addition to tortuosity, each line in the Voronoi network was assigned a value representing the vessel radius. This value was drawn from a Gaussian distribution with a mean and standard deviation selected based on histological data; here for the human primary visual and motor cortex with a mean of 3.235 µm and a standard deviation of 0.850 µm [57,66], and the mouse somatosensory cortex with a mean of 1.750 µm and a standard deviation of 0.300 µm [45]. Other cortical regions or species can be simulated by selecting distribution parameters derived from the corresponding histological data. Moreover, the synthetic capillary network maintains radius continuity across adjacent segments. During the generation of the 3D VAMOS network, we implemented a local “smoothing” constraint ensuring that neighboring vessel segments are assigned similar radii. This approach avoids unphysiological, abrupt changes in vessel diameter at bifurcations or along continuous paths, thereby ensuring that the morphological properties of the capillary bed remain consistent and biologically plausible.

#### Generation of the macrovascular architecture based on kernel functions.

Building upon the predefined three-dimensional space for the microvascular network, the macrovascular architecture was subsequently constructed. The process begins with generating the pial arteries and veins, whose quantity is determined by the predefined initial number of penetrating arteries and ascending veins, based on literature values for a particular cortical region. For the human brain, the artery-to-vein ratio was set to approximately 3:1 [51], corresponding to 42 penetrating arteries and 14 ascending veins for the primary visual cortex, and 85 penetrating arteries and 25 ascending veins for the primary motor cortex [57,66]. For the mouse barrel cortex 5 penetrating arteries and 15 ascending veins were set [45]. At the top plane of the volumetric space in the z-direction (i.e., the maximal z-cross-section), seed points were randomly placed in the xy-plane, constrained by a predefined minimum “proximity distance” between penetrating and ascending vessel seed points, set to ~120 μm [51]. Each seed point was then designated as either an artery or a vein. This designation followed the principle that veins in humans must be surrounded by arteries: if a seed point was designated as a vein, its nearest neighbors were preferentially labelled as arteries, consistent with the histological artery-to-vein ratios [51,64]. For the mouse models the artery-to-vein ratio was reversed [45]. Next, the labelled pial artery seed points at the maximal z-cross-section were interconnected using kernel functions, with vessel tortuosity fixed at ~1.25[-]. The same process was applied to the labelled pial veins (see Fig 2).

After creating the pial vasculature, the subsequent step generated the main branches of the penetrating arteries and ascending veins. The definition of the cortical penetration depth for these vessels was based on Duvernoy et al. [51]. Penetrating arteries or ascending veins were classified into five cortical depth equidistant bins, as Artery (A1-A5), or Vein (V1-V5) from the pial surface (A1/V1) to the GM-WM boundary (A5/V5 (see Fig 2). Each endpoint of a penetrating vessel was aligned parallel to its corresponding surface seed points, i.e., at the maximal z-cross-section, and subsequently connected by another set of kernel functions.

Furthermore, the number of sub-branches, or daughters of the main vessel segment, can be predetermined as an initial parameter for each penetrating or ascending vessel. These sub-branches were randomly positioned along the main vessel and generated using a set of kernel functions. The vessel radius of both the penetrating or ascending vessels and their sub-branches follows a branching exponent adhering to Murray’s law [67,68]:


$$\begin{array}{c} {\text{R}}_{\text{parent}}^{\text{k}}={\text{R}}_{\text{daughter1}}^{\text{k}}+{\text{R}}_{\text{daughter2}}^{\text{k}} \end{array}$$ (2)

with k ranges between 2 and 3 in both humans and rodents; here we selected k = 2. While Murray’s law typically utilizes an exponent of k = 3 [67], we utilized an exponent of k = 2 for the 3D VAMOS framework. This choice follows the area-preservation law, which dictates that the total cross-sectional area remains constant across a bifurcation. From a physiological perspective, an exponent of k = 2 is characteristic of larger vessels where it is necessary to maintain constant blood flow velocity and minimize pulse wave reflections [68]. From a computational and modelling standpoint, utilizing k = 2 serves as a robust simplification that prevents vessel radii from tapering too aggressively as the network depth increases. This ensures a stable and physiologically plausible CBV across the simulated volume, avoiding the ‘thinning’ artifacts that can occur in complex synthetic trees when k = 3 is applied strictly to every microvascular junction. Radii values were initialized at the pial surface seed points (at the maximal z-cross-section), and gradually decreased across cortical depth, reaching their smallest size at the endpoints of the main branches and their sub-branches (see Fig 2). For the human models, intracortical vessel radii ranged from 13 µm to 23 µm for arteries, and 15.65 µm to 31.65 µm for veins [57,66]. For the mouse models, intracortical vessel radii ranged from 7 µm to 12 µm for arteries, and 10 µm to 15 µm for veins.

Consequently, the 3D VAMOS algorithm currently generates for each parent penetrating or ascending vessel (main branch) a specified number of daughter vessels expanding in a radial pattern (sub-branches), resembling a topological tree-like structure [54]. The number of sub-branches, their length, and tortuosity can be set to different values dependent on the cortical region simulated.

Another feature of 3D VAMOS is the option to connect V5 ascending veins at the GM-WM border. When this parameter is enabled, all V5 veins are interconnected at their endpoints by using a set of kernel functions. The inclusion of connections between V5 veins (large intracortical veins) is motivated by the known presence of deep venous plexuses and collateral connections at the gray-white matter border. Anatomical studies of the human cerebral cortex have identified principal intracortical veins (PICVs) that serve as the primary drainage route for deep cortical layers [63,90,101]. These vessels often exhibit horizontal anastomoses or connections at deeper laminar depths, which facilitate the efficient transport of deoxygenated blood from the deep parenchyma toward the pial surface. By including these connections, 3D VAMOS accurately represents the ‘sink’ effect of the venous system, which is crucial for modelling the spatial specificity of the BOLD signal and the influence of large-vessel ‘blooming’ artifacts across cortical depth.

#### Physical connection between vascular compartments.

After generating both the macro- and micro-vasculature, all the endpoints of the macrovascular branches and sub-branches were connected to the nearest junction of the microvascular network using the shortest Euclidean path, resulting in a fully interconnected cerebrovascular network.

### 4.2 Simulation of the BOLD signal using the 3D VAMOS accounting for intravascular and extravascular signal contributions

In this study, BOLD fMRI signals consisted of both extravascular and intravascular signal components influenced by the vascular architecture, baseline CBV (see Fig 1), oxygen saturation (SO_2_) in vessels, and hematocrit. The total MRI signal,  S(t), was calculated by summing the extravascular signal contribution, $S_{EV}$, with both arterial, $S_{aIV}$, and venous, $S_{vIV}$, intravascular signal contributions:


$$\begin{array}{c} S(t)=\;S_{EV}(t)+S_{aIV}(t)+S_{vIV}(t) \end{array}$$ (3)

Simulations were performed for gradient-echo (GE) and spin-echo (SE) pulse sequences at seven tesla (7T), assuming an instantaneous readout, i.e., without spatial encoding such as echo-planar-imaging (EPI). Additionally, we assumed that the cortical surface normal of the models was parallel to the main magnetic field $B_{\text{0}}$ [10,47,50,92].

#### Arterial and venous intravascular signal contributions.

The intravascular signal contribution for each vascular compartment was approximated as follows:


$$\begin{array}{c} S_{aIV}(t)=\left(b{CBV}_{a}\right)\cdot \left(e^{-{{R\text{2}}^{*}}_{dHb}\cdot t}\right)\;for\;GE;\;and\;S_{aIV}(t)=\left(b{CBV}_{a}\right)\cdot \left(e^{-{R\text{2}}_{dHb}\cdot t}\right)\;for\;SE \end{array}$$ (4)

and


$$\begin{array}{c} S_{vIV}(t)=\left(b{CBV}_{v}\right)\cdot \left(e^{-{{R\text{2}}^{*}}_{dHb}\cdot t}\right)\;for\;GE;\;and\;S_{vIV}(t)=\left(b{CBV}_{v}\right)\cdot \left(e^{-{R\text{2}}_{dHb}\cdot t}\right)\;for\;SE \end{array}$$ (5)

Where t is time, $b{CBV}_{a}$ and $b{CBV}_{v}$ are baseline arterial and venous blood volume fractions respectively (see Fig 1 for representative bCBV’s), and the relaxation rates ${{R\text{2}}^{*}}_{dHb}$ and ${R\text{2}}_{dHb}$ for GE and SE, respectively, are given by:


$$\begin{array}{c} {{R\text{2}}^{*}}_{dHb}=\;{{R\text{2}}^{*}}_{\text{0},in}+{R\text{2}}_{SO\text{2}}\;for\;GE;\;and\;{R\text{2}}_{dHb}=\;{R\text{2}}_{\text{0},in}+{R\text{2}}_{SO\text{2}}\;for\;SE \end{array}$$ (6)

where, ${{R\text{2}}^{*}}_{\text{0},in}$ or ${R\text{2}}_{\text{0},in}$, are the intrinsic relaxation rates of blood, and ${R\text{2}}_{SO\text{2}}$ the relaxation rate dependent on SO_2_.

We assumed that the arterial and venous intravascular signal contribution to the BOLD signal is non-zero. This assumption is based on the observation that at high magnetic fields strengths, the intravascular arterial and venous signals, particularly in the veins, are not negligible at higher (≥ 80%) SO_2_ levels [31,34,102]. On the other hand, we assumed the intravascular signal contribution of the microvascular compartment to be null, given that the ${{R\text{2}}^{*}}_{dHb}$ and ${R\text{2}}_{dHb}$ of the capillaries has not been well-characterized, and the intravascular contribution of capillaries is not expected to be as significant as that of larger vessels [31].

Therefore, for GE we calculated ${\text{1}/{R\text{2}}^{*}}_{\text{0},in}={{T\text{2}}^{*}}_{\text{0},in}$ (≈ 10.00 ms), and ${R\text{2}}_{SO\text{2}}$ component dependent on SO_2_ using the linear (Eq. 7 in [32]) and quadratic (Eq. 8 in [32]) relationships, respectively (see Tables 1 and 2), weighted by the corresponding blood volume fraction (see Fig 1). For SE we calculated ${\text{1}/R\text{2}}_{\text{0},in}={T\text{2}}_{\text{0},in}$ (≈ 53.82 ms), and ${R\text{2}}_{SO\text{2}}$ component dependent on SO_2_ [32] (see Tables 1 and 3).

**Table 1:** Biophysical and pulse sequence parameters used to compute the relaxation rates and BOLD signal changes.

biophysical and MR pulse sequence parameters	
diffusion coefficient (extravascular tissue)	1.0 µm^2^/ms; isotropic motion across cortical depth
static magnetic field strength	7 T
Systemic hematocrit (Hct)	45%
echo-time (TE)	GE = 27 ms SE = 50 ms
time-step	0.020 ms
number of spins	30 Monte-Carlo repetitions with 5 ∙ 10^7^ spins for each repetition
GE: T2_0_* cortical gray matter at 7T SE: T2_0_ cortical gray matter at 7T	28.57 ms 48.30 ms
GE: intravascular arterial T2*_dHb_ at 7T	9.87 ms @ SO_2_ = 95%
SE: intravascular arterial T2_dHb_ at 7T	49.67 ms @ SO_2_ = 95%
GE: intravascular venous T2*_dHb_ at 7T	see Table 2.
SE: intravascular venous T2_dHb_ at 7T	see Table 3.

**Table 2:** GE: intravascular venous T2_dHb_* at 7T.

SO_2_ [%]	60	62.5	65	67.5	70	72.5	75	77.5	80
T2*_dHb_ [ms]	5.40	5.78	6.17	6.59	7.01	7.45	7.89	8.02	8.31

**Table 3:** SE: intravascular venous T2_dHb_ at 7T.

SO_2_ [%]	60	62.5	65	67.5	70	72.5	75	77.5	80
T2_dHb_ [ms]	8.13	9.23	10.56	12.17	14.12	16.51	19.44	22.43	23.03

#### Implementation of vessel-dependent oxygen saturation levels.

We simulated nine different SO_2_ levels per vascular compartment, which were maintained constant over time, i.e., steady-state SO_2_ levels were assumed [103]. The SO_2_ values used in the microvessels depended on the SO_2_ imposed on the veins, as follows:

- SO_2_ in veins (SO_2_vein) = [60%, 62.5%, 65%, 67.5%, 70%, 72.5%, 75%, 77.5%, 80%].
- SO_2_ in microvessels = SO_2_art - ((SO_2_art - SO_2_vein)/ 2);
- SO_2_ in arteries (SO_2_art) = 95%;

An arterial SO_2_ 95% was chosen as the minimal physiological value in that compartment. Increments in the steady-state arterial SO_2_ value, within the range of 96% to 100%, would likely reduce the arterial contribution to the MR signal formation. Venous SO_2_ values ranged from 60% during the resting state to 80% during the active state, within physiologically plausible values [28,29]. The SO_2_ results in Figs 4 and 5 use solid lines with dots to denote the mean values across the various oxygen saturation scenarios, with shaded regions representing the standard deviation computed across several Monte Carlo simulations.

#### Implementation of vessel size-dependent hematocrit level.

The formation of the BOLD fMRI signal is fundamentally influenced by systemic hemoglobin concentration, i.e., hematocrit (Hct), and variations in Hct between the different vascular compartments may induce differences in cortical depth-dependent BOLD fMRI signal changes [9]. In this study, we simulated a vessel size-dependent Hct level using experimental values obtained by Gould et al., [2017] as follows:

- arteries: 0.90 * Hct;
- microvessels: 0.70 * Hct;
- veins: 1.20 * Hct,

The weighting value (1.20) for the veins was chosen to represent a plausible extreme scenario in which red blood cells accumulate, leading to a stalling effect in this vascular compartment, particularly in the venules. Since the BOLD fMRI signal is highly sensitive to venous contributions, and the simulated microvasculature does not clearly distinguish between arterioles, small capillaries, and venules, we simulated this effect in the larger venous compartment as a proxy for its impact on the BOLD fMRI signal [Gould et al., 2017]. Reduced weighting values in the veins would likely only decrease their contribution to the fMRI signal [31].

From here onwards, we used the systemic Hct definition to set the values in the simulations, ensuring that the Hct value always corresponded to the vessel size characteristics as defined previously.

#### Simulation of the extravascular signal contribution.

The extravascular signal contribution $S_{EV}(t)$ for both macro- and micro-vessels was computed by modelling the interaction of moving spins within the local inhomogeneous magnetic field induced by the varying SO_2_ levels [34,102].

The local frequency shift caused by a vessel segment was modeled as the dipolar induced-response of an infinite cylinder, presuming negligible effects on the cylinder extremities [38,50], given by (in [1/s]):


$$\begin{array}{c} \delta \omega (r)=\frac{\text{1}}{\text{2}}\cdot \frac{\gamma }{\text{2}\pi }\cdot B_{\text{0}}\cdot \Delta \chi \cdot \left(\frac{R^{\text{2}}}{r^{\text{2}}}\right)\cdot \text{cos}(\text{2}\theta )\cdot {sin}^{\text{2}}\psi \end{array}$$ (7)

where γ is the hydrogen gyromagnetic ratio = 267.5x10^6^ [rad∙(s·T)^-1^], $B_{\text{0}}$ is the main magnetic field (7 [T]), $\Delta \chi =\text{4}\pi \cdot \text{0}.\text{2}\text{7}\text{6}\;ppm\cdot HcT(R)\cdot \left(\text{1}-{SO}_{\text{2}}\right)$ [-] is the susceptibility difference produced by the ${SO}_{\text{2}}$ in the vessel [39] and the vessel size-dependent hematocrit Hct(R) [104], R is the vessel radius in [µm], r is the Euclidean distance from the center line of the vessel to a particular spatial position in the simulation volume in [µm], θ is the angle between the vessel and the spatial position in [rad], and ψ is the angle between the orientation of the vessel and the main magnetic field in [rad].

The dephasing experienced by a population of diffusing spins, $N_{spins}$, was simulated using a Monte Carlo approach with 30 repetitions per SO_2_ state, and a diffusion coefficient of D = 1.0 [µm^2^ ∙ ms^-1^], assuming isotropic diffusion across cortical depth [37]. In each repetition, a new 3D VAMOS model was generated (30 repetitions x 9 SO_2_ states = 270 models), and 5x10^7^ spins were simulated. Spin dephasing was obtained through:


$$\begin{array}{c} \varphi (t)=\int_{\text{0}}^{t}\delta \omega \left(x(t)\right)\;dt \end{array}$$ (8)

where φ(t) is the phase acquired during the simulation time t and δω(x(t)) is the local frequency shift at spin position x at each time-step t (= 0.020 ms). For SE sequences, the acquired dephasing after TE/2 was multiplied by -1 (change in polarity) simulating the effect of the 180-degree refocusing radiofrequency pulse. Using equation (9) we can obtain the extravascular MR signal contribution as follows:


$$\begin{array}{c} S_{EV}(t)=\left(\text{1}-\left({bCBV}_{a}+{bCBV}_{v}\right)\right)\cdot \left[S_{R{\text{2}}^{\prime }}\cdot e^{-{{R\text{2}}^{*}}_{\text{0}}\cdot t}\right]\;for\;GE; \end{array}$$

$$\begin{array}{c} {\;S}_{EV}(t)=\left(\text{1}-\left({bCBV}_{a}+{bCBV}_{v}\right)\right)\cdot \left[S_{R{\text{2}}^{\prime }}\cdot e^{-{R\text{2}}_{\text{0}}\cdot t}\right]\;for\;SE \end{array}$$ (9)

where $S_{R{\text{2}}^{\prime }}$ represents the MR signal component induced by the interaction of diffusing spins within the local magnetic field inhomogeneities (i.e., the signal subject to $R{\text{2}}^{\prime }$ decay). This signal component depends on the specific pulse sequence and is defined as,


$$\begin{array}{c} S_{R{\text{2}}^{\prime }}=\left(\frac{\text{1}}{N_{spins}}\sum^{N_{spins}}e^{-i\varphi (t)}\;\right) \end{array}$$ (10)

and ${{R\text{2}}^{*}}_{\text{0}}$ = 1/T2_0_* and ${R\text{2}}_{\text{0}}$ = 1/T2_0_ are the intrinsic relaxation rate of cortical tissue for GE and SE, respectively. We used the intrinsic tissue T2_0_* ≈ 28 ms for GE and T2_0_ ≈ 48 ms for SE according to the nonlinear relationship given by Khajehim et al., [105] for gray matter at 7T (see Table 1).

To confine the ensemble of spins within the simulation space, voxel boundary conditions were set to infinite space. Spins exiting the voxel re-entered the voxel volume on the opposite side, preserving their dephasing history. However, spins reaching the pial surface and GM-WM border were considered invalid iterations and were recomputed. Additionally, spin exchange between extra/intra-vascular compartments was prohibited, establishing an impermeable vascular network [32].

#### Relaxation rates and BOLD signal changes.

Given that the behavior of S(t) in (Eq. 2) presents oscillations due to its multi-exponential nature, we simply approximated the global relaxation rate ${R\text{2}}^{*}$ and R2 by fitting a polynomial of degree one, i.e., a linear fit, on the natural logarithm of S(t), i.e., ${R\text{2}}^{\left(*\right)}=\frac{\text{ln}\left(S(t)\;\right)}{t}$, for GE and SE, respectively. In this work, two metrics are reported:

(1) the relative relaxation rate ${\Delta R\text{2}}^{\left(*\right)}$ in [1/s]:,


$$\begin{array}{c} {\Delta R\text{2}}^{\left(*\right)}=\;\left(\frac{{{R\text{2}}^{\left(*\right)}}_{SO\text{2}}}{{{R\text{2}}^{\left(*\right)}}_{tissue}}-\text{1}\right) \end{array}$$ (11)

where ${{R\text{2}}^{\left(*\right)}}_{SO\text{2}}$ represents the varying SO_2_ states, and ${{R\text{2}}^{\left(*\right)}}_{tissue}$ is the intrinsic relaxation rate of tissue and (2) the relative BOLD signal change, ΔBOLD in [%]:,


$$\begin{array}{c} \Delta BOLD=\;\left(\frac{{S(TE)}_{SO\text{2}}}{{S(TE)}_{tissue}}-\text{1}\right)\times \text{1}\text{0}\text{0} \end{array}$$ (12)

where ${S(TE)}_{SO\text{2}}$ represents the varying SO_2_ states, and (${S(TE)}_{tissue}=\text{exp}(-{{R\text{2}}^{\left(*\right)}}_{tissue}\cdot TE)$) is the tissue signal at TE and is defined as the baseline condition for GE and SE respectively.

#### Using random distributed oriented cylinder (RADOC) models to simulate cortical depth-dependent BOLD signals.

In order to demonstrate the advantages of using a synthetic 3D vascular model, we generated a composite vascular model using RADOCs, as displayed in Fig 2, to simulate the cortical depth-dependent BOLD contribution in simplified, non-realistic vascular models (see Fig 5). Macrovascular RADOCs were simulated as cylinders with radii ranging from 10 µm to 40 µm, and microvascular RADOCs as cylinders with radii of 1 µm to 6 µm (Fig 2). Both compartments were assigned cortical depth-dependent volume fractions within a cortical slab of 1 mm thickness, divided it into eight equidistant laminae. The macrovascular volume fraction was set to 4% at the cortical surface, decreasing linearly to 1% at the deepest laminae. The microvascular volume fraction followed a Gaussian distribution, peaking at 1% in the middle laminae and slightly decreasing toward the cortical surface and the GM-WM border. SO_2_ levels ranged from 60% to 80%, representative of venous oxygenation, and biophysical properties of tissue were as described previously [31]. Furthermore, to control the cerebral blood volume across specific cortical lamina in the RADOC model, we defined a spatially-constrained simulation domain for each lamina. The in-plane dimensions of this domain were set to several hundred micrometers—a scale significantly larger than the maximal water diffusion path—to ensure that the BOLD signal contribution is statistically representative and free from boundary artifacts. Within this defined volume, cylindrical segments are iteratively populated with orientations and vessel radius drawn from a random distribution. The algorithm continues adding these segments until the target CBV for that specific laminar depth is achieved. While the cylinders are modeled as ‘infinite’ to avoid unphysiological end-cap effects (which could introduce artificial magnetic susceptibility gradients), their contribution to the total volume fraction is strictly calculated based on their intersection with the defined laminar boundaries (simulation domain). This ensures that the CBV is precisely controlled for each lamina independently, regardless of the vessel length.

### 4.3 Simulating neurovascular effects with phenomenological approaches

To investigate neurovascular responses within this synthetic vascular environment, we employed a phenomenological modelling strategy based on Grubb’s law [106], which characterizes the empirical relationship between cerebral blood flow (CBF) and cerebral blood volume (CBV). This relationship is given by:


$$\begin{array}{c} CBV={CBF}^{\alpha } \end{array}$$ (13)

where α is typically ~0.38 for cortical gray matter [106], but recent studies suggest smaller values that better capture hemodynamic behavior across cortical depth. We adopted α = 0.25, based on the rationale that, according to Poiseuille’s law—which relates blood flow changes to vessel radius—the radius scales with a fourth-power law. This choice is further supported by findings from Ito et al. [107] and Chen & Pike [108], which better reflect cortical depth-dependent vascular dynamics at the mesoscale [107,108].

In our simulations, CBF changes were modeled as a surrogate for neuronal activity. Global increases in CBF were simulated within a range of 0% to 40% relative change and applied uniformly across all 3D VAMOS vessels. Corresponding CBV changes were then computed using the established power-law relationship [109], which uniformly alters the CBV of all vessels, regardless of vessel type. For example, a vessel with a radius of 10 μm undergoing a 20% relative increase in CBF would exhibit a ~ 4.6% relative change in CBV, resulting in a final radius of approximately 10.46 μm.

To mimic localized neuronal activation, we structured the simulations into six conditions, comprising three cortical depth locations and three vessel-specific hemodynamic response types. Simulated neuronal activity was positioned at superficial (0.35 mm), middle (1.0 mm), and deep (1.5 mm) cortical depths. For each location, vessel-specific changes in CBV and SO_2_ were modeled under three conditions: **(1)** capillaries and veins dilated locally, with CBV varying between 0–30%, and arterial and capillary SO_2_ changing locally within the same range; **(2)** only capillaries dilated locally, with both CBV and SO_2_ each varying between 0–30% at the respective activity site; and **(3)** all vessel types dilated locally, with both CBV and SO_2_ changing from 0–30% at the corresponding location (see Fig 6 and S1 Appendix). No permutations between CBV and SO_2_ changes were included. The hemodynamic point spread function was modelled with a Gaussian spatial profile with a of 0.150 mm [73,110]. For instance, consider a vessel with an initial radius of 10 μm. A global 20% increase in CBF would increase its radius to approximately 10.46 μm. If, in addition, a 10% local increase in CBV is applied (reflecting the neuronal activation intensity), the final vessel radius would reach about 11.50 μm.

Simulations were performed with 3D VAMOS models of the human primary visual cortex, using baseline hemodynamic conditions, including flow rates and SO_2_ levels, from the literature [73,74,110]. BOLD signal changes were computed relative to a baseline condition of CBF = 0% across cortical depth, thereby isolating the BOLD effect associated with the simulated neuronal activity (see Fig 6 and S1 Appendix). This phenomenological approach allowed to generate neurovascular responses without explicitly modelling neuronal activity.

## Supporting information

S1 Appendix Complete simulation results of neurovascular effects based on phenomenological modelling. (DOCX)

## Abbreviations:

3D: three-dimensional
7T: 7 tesla
BOLD: blood oxygenation level-dependent
CBF: cerebral blood flow
CBV: cerebral blood volume
CMRO2: oxygen metabolism
CSF: cerebrospinal fluid
fMRI: functional magnetic resonance imaging
GE: gradient echo
GM: gray matter
HcT: hematocrit
SE: spin echo
SO_2_: oxygen saturation
TE: echo time
VAMOS: vascular model based on statistics
WM: white matter
