# Supplementary material for: A synthetic 3D human cerebrovascular model informed by histology for simulating the cortical depth-dependent BOLD fMRI signal
Source: PLoS Comput Biol. 2026 Aug 3;22(8):e1014576. doi: 10.1371/journal.pcbi.1014576 (PMC13450845; doi:10.1371/journal.pcbi.1014576)
Supplement: Supplementary file 1 [file pcbi.1014576.s002.pdf]

Reviewer 1

1. Pg. 42, Eq. (7): Using the infinite cylinder model for the field offsets around a finite vessel segment is highly questionable and seems like only a matter of computational convenience, rather than what is biophysically accurate.

- How do the authors treat a spin that lies along the central axis of a vessel but is just outside the segment?
- The tortuosity of the capillaries would completely invalidate the assumptions being made.
- There are field offsets around the vessel ends and where vessels intersect that are not well-represented by the infinite cylinder model.

R: We thank Reviewer 1 for raising this important point regarding the use of the infinite cylinder approximation for computing magnetic field perturbations.

We would like to clarify that this approach is not merely a matter of computational convenience, but rather a well-established approximation that has been extensively used in the MRI literature [e.g., Bieri & Scheffler, 2007; Chaussé et al., 2025; Aghaeifar et al., 2025; Genois et al., 2021; Khajehim et al., 2017; Uludağ et al., 2009] to model susceptibility-induced field inhomogeneities around blood vessels (e.g., Ogawa et al., 1993; Yablonskiy and Haacke, 1994; Boxerman et al., 1995), and forms the basis of many biophysical models of the BOLD signal.

Its validity relies on the fact that, at the spatial scale relevant for spin diffusion and signal formation, vessel segments can be locally approximated as straight cylinders. In our manuscript, a tortuous vessel is represented by a number of small straight cylinders. Thus, in the next Figures, we demonstrate the viability of computing inhomogenous magnetic fields for two different vascular models published and peer-reviewed in two different top-tier journals.

In the following figure, we show the inhomogeneous magnetic field created by the realistic mouse vascular model. This result and its corresponding laminar-dependent BOLD signal changes were published in Báez Yáñez et al., 2025. The local inhomogeneities displayed differences at the top and middle planes, where the vascular compartments differ in vessel type and density.

From: **Impact of vascular architecture, oxygen saturation, and hematocrit on human cortical depth-dependent GE- and SE-BOLD fMRI signals: A simulation approach using realistic 3D vascular networks**

. 2025;3. doi:10.1162/imag_a_00573

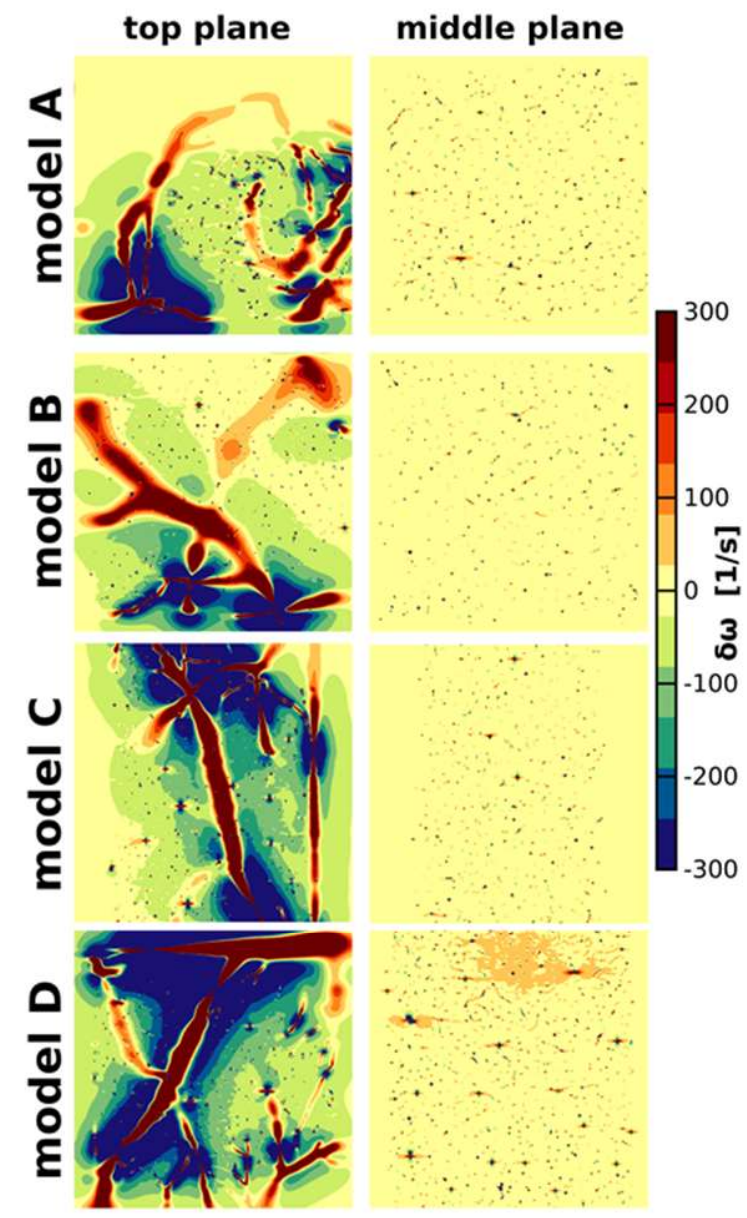

Figure Legend:

Local frequency field distortions produced by the realistic vascular models for an exemplary oxygen saturation level (SO_2vein = 78%) and hematocrit (Hct = 45%) are shown. The oxygen saturation value was arbitrarily selected for illustration purposes. The frequency field maps are depicted for two different locations inside the simulated voxel: the top plane, situated approximately 20 µm deep from the cortical pial surface, and the middle plane, positioned around 500 µm deep relative to the pial surface. The main magnetic field was simulated to be orthogonal to the cortical surface and thus to the depicted planes.

Date of Download: 4/2/2026

Copyright © 2026. MIT Press. All rights reserved.

In the following figure, we demonstrate the computation of the inhomogenous magnetic field for a statistical vascular model where the large vessels were extracted from real vascular data and superimposed on the synthetic capillaries.

A mechanistic computational framework to investigate the hemodynamic fingerprint of the blood oxygenation level-dependent signal

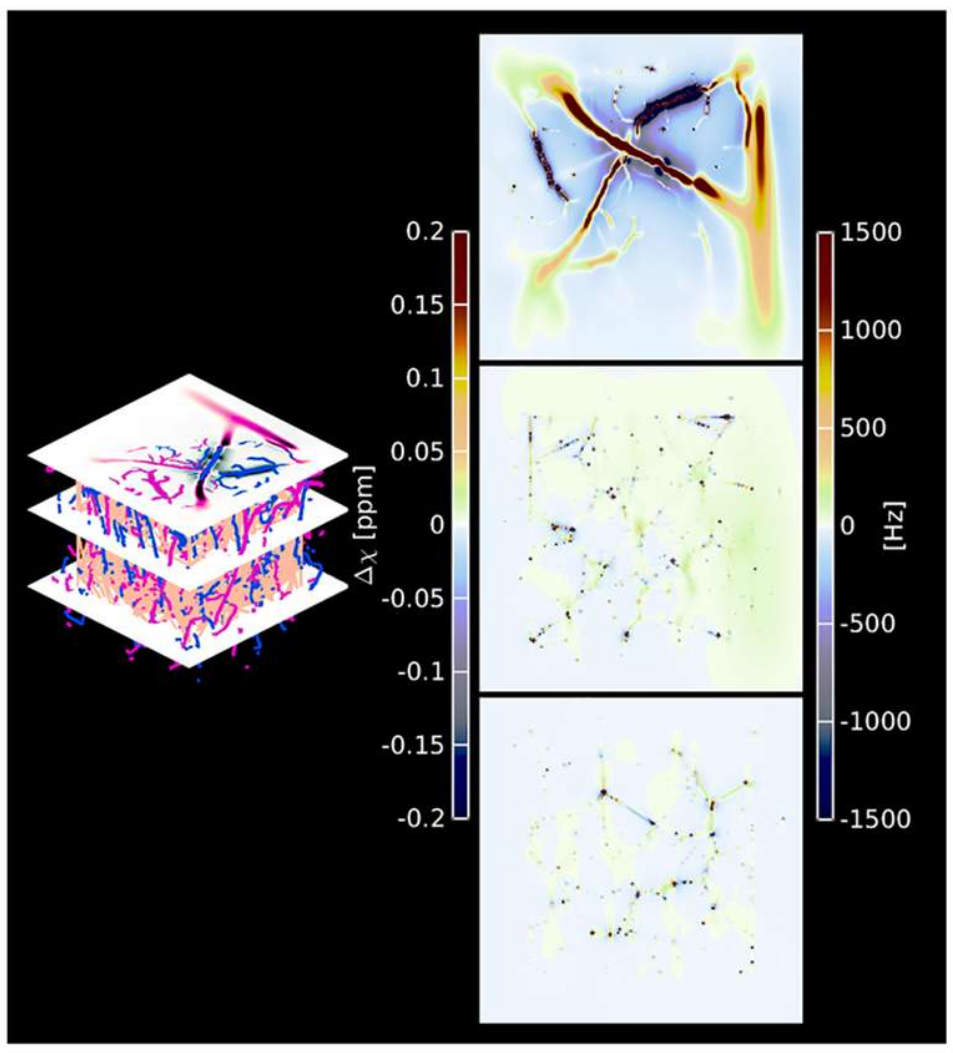

NMR in Biomedicine, Volume: 36, Issue: 12, First published: 29 August 2023, DOI: (10.1002/nbm.5026)

Further we include a validation-type result were we explain the cortical orientation amplitude-dependence of the BOLD fMRI signal as reported in experimental data from Fracasso et al., 2018; Viessmann et al., 2019; Gagnon et al., 2016. These results were peer-reviewed and published in Báez Yáñez et al., 2023.

A mechanistic computational framework to investigate the hemodynamic fingerprint of the blood oxygenation level-dependent signal

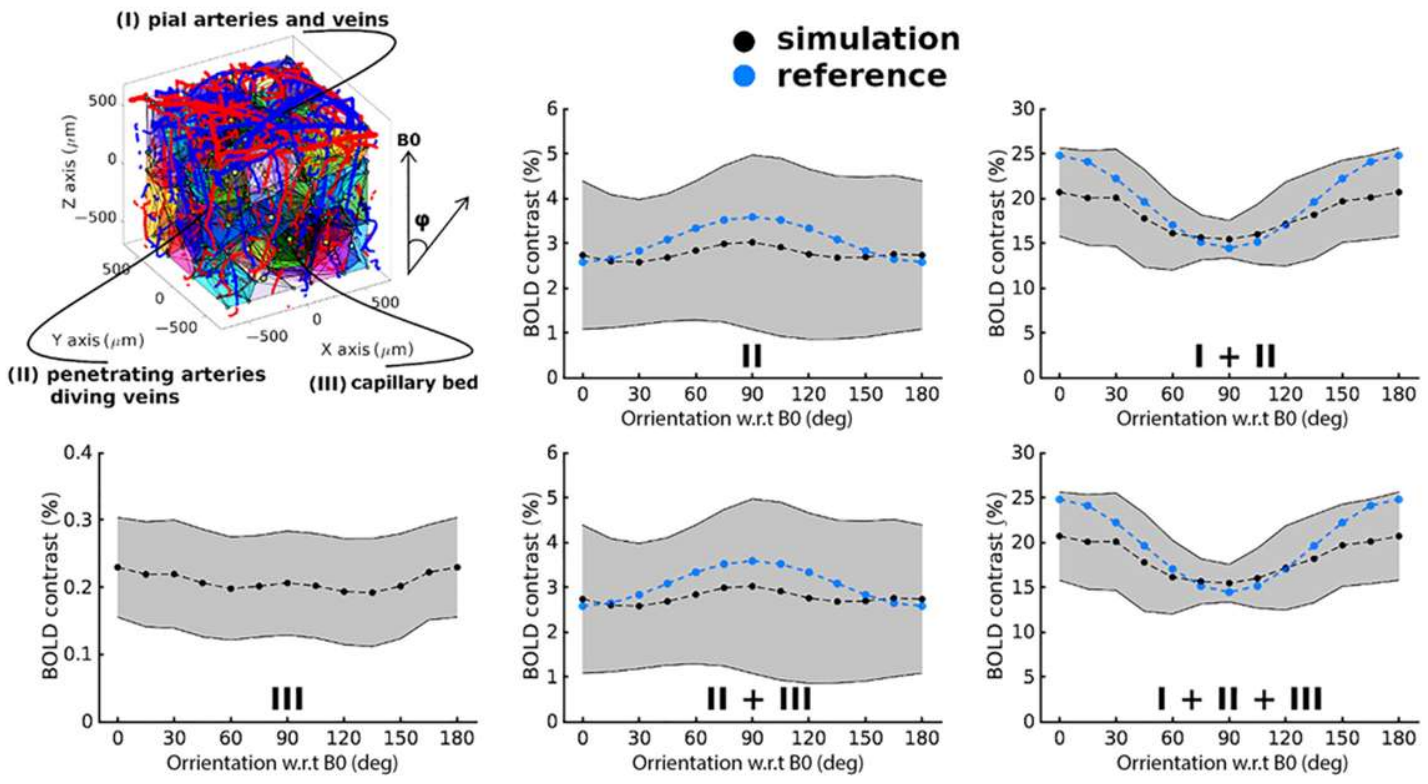

NMR in Biomedicine, Volume: 36, Issue: 12, First published: 29 August 2023, DOI: (10.1002/nbm.5026)

While we fully agree that real vessels are finite, tortuous, and form complex branching networks, the infinite cylinder model provides an accurate local approximation of the magnetic field for length scales where the vessel segment can be considered locally straight relative to the diffusion length of the spins. In particular, the BOLD signal is predominantly influenced by mesoscopic field variations averaged over diffusive spin trajectories, which reduces sensitivity to localized geometric features such as vessel ends or branching points.

Regarding the specific concern about spins located near vessel endpoints or along the axis of finite segments, we note that these configurations represent a small fraction of the total spatial domain and contribute negligibly to the overall signal due to spatial averaging inherent in the Monte Carlo simulation framework, which utilizes approximately $1x10^7$ iterations to ensure statistical convergence. Furthermore, the stochastic nature of the vascular network and spin sampling mitigates systematic biases arising from such localized deviations. Importantly, the validity of this approximation in the context of BOLD signal modelling has been demonstrated in multiple prior studies, including our own previous work [Báez Yáñez et al., 2017; Báez Yáñez et al., 2023; Báez Yáñez et al., 2025; van Horen et al., 2023], where we showed that the resulting signal characteristics are consistent with both theoretical predictions and experimental observations.

Therefore, we respectfully argue that the use of the infinite cylinder approximation is justified, well-supported by the literature, and appropriate for the scale and objectives of the present study.

The reviewer’s concern appears to stem from an interpretation that this approximation invalidates the results; however, based on existing evidence and prior validation, we believe this conclusion is not warranted. Furthermore, we

acknowledge that the infinite cylinder model is an approximation and does not explicitly account for field perturbations at vessel ends or intersections. To address this point, we have included an expanded discussion in the manuscript clarifying the assumptions, limitations, and domain of validity of this approximation.

In light of the above, we respectfully suggest that the reviewer's concern reflects a limitation inherent to many established BOLD modelling frameworks rather than a fundamental flaw in the methodology. We therefore believe that this point does not invalidate the results or conclusions of the present study.

2. Pg. 7, line 158: The range of capillary radii are taken from ex vivo data that likely underestimated the actual radii. Using radii as small as 1 um does not seem physiologically plausible, particularly given the size of a RBC.
Can the authors comment more on why such small radii were used.
R: Regarding the use of small capillary radii (down to 1 µm), we acknowledge that histological measurements can be influenced by tissue shrinkage during processing, potentially underestimating in-vivo dimensions. However, these values provide a consistent statistical foundation for our model. Furthermore, the inherent deformability of red blood cells allows for passage through microvessels with diameters significantly smaller than their resting state [Gould et al., 2015], supporting the physiological relevance of including these smaller scales in our simulations.

3. Pg. 8 line 177: Regarding Fig 1.C and 1.D, the authors write, "Each graph shows thirty superimposed distributions, illustrating the anatomical consistency of models generated…" The reproducibility is challenging to discern from the figures since the lines just look like a single thick line, particularly for the radius distributions.
It may be easier to visualize if the lines were slightly different colours or shades, or if a single line for the mean/median was used plus shaded bars representing the range or standard deviation.
Additionally, can you please provide a legend for the colour coding in the radius distributions.
R: Thank you for the suggestion. The display of the results in Fig.1.C and Fig.1.D has been updated accordingly.

4. Pg. 11, line 223:
Why were uniform distributions for the ROC model chosen and not something more similar to what was used in the VAMOS networks? This would make comparing the simulations from the two more comparable.
R: The reviewer makes an excellent point regarding the comparability of the models. We chose uniform distributions for the RADOC (previously ROC) model specifically to serve as a benchmark as used in traditional biophysical modelling approaches, which historically rely on simplified, uniform vascular geometries. By maintaining this standard configuration for RADOC, we can more clearly isolate and quantify the specific impact that the biologically informed vessel distributions (used in 3D VAMOS) have on BOLD fMRI signal generation. Our results demonstrate that the transition from these simplified uniform assumptions to the more realistic, heterogeneous distributions provided by 3D VAMOS is essential for capturing nuanced laminar BOLD profiles. We have now clarified this point in Pg.9.

5. Pg. 15, Fig. 3:
Why are there no visible surface arteries in the realistic mouse model (A)?
The match between the large radii distributions from 10-12 um in D.

Similarly, the venous volume fraction matches seem quite off in H (veins don't seem very present in the realistic model).
R: We appreciate the reviewer's careful observation regarding the differences between the realistic mouse model and our synthetic framework. These discrepancies primarily stem from the vascular classification methodology used in the reference dataset: (1) Surface arteries and large vessels (A & D): As discussed in the manuscript, vessel labelling in the realistic model [Báez-Yáñez et al., 2025] was performed using manual classification based on radius thresholds. This approach can lead to an underrepresentation of surface vessels if they do not meet specific threshold criteria or if labelling was restricted to parenchymal volumes. (2) Venous volume fraction (H): The apparent lack of veins in the realistic model is a known limitation of threshold-based segmentation. Because the classification was not based on physiological markers, but rather on geometric heuristics, certain venous compartments may have been misclassified or omitted. These factors directly influence the calculated compartment volume fractions. However, by using these realistic datasets as a benchmark, we highlight that 3D VAMOS's ability to bridge these gaps by generating statistically consistent networks based on known histological parameters.

6. Pg. 16, starting at line 295: Going from vascular architecture to BOLD signal (i.e., Fig. 3 to Fig. 4) is quite the leap. In between one must have some distribution of blood flow and oxygenation across the vessels.
How were these computed and how did they compare?
R: We agree that the transition from vascular architecture to BOLD signal depends on the underlying physiological state. We have clarified this transition in the revised manuscript by adding a paragraph to the Results section (pg. 15, line 294) to remind the reader of our modelling approach. Specifically, the objective of this study was not to simulate dynamic, time-dependent hemodynamic changes, but rather to establish a robust framework for mapping static vascular architecture to BOLD signal contributions. To achieve this, we assigned physiologically plausible, steady-state hemodynamic parameters, such as blood oxygenation and volume, to each vascular compartment based on established literature values for baseline and activated states. This approach allows us to isolate the influence of vascular geometry on the BOLD signal without the added confounding variables of dynamic flow modelling. These parameters and their implementation are described in detail in the Methods (see Implementation of vessel-dependent oxygen saturation levels and Implementation of vessel size-dependent hematocrit level).

7. Pg. 24, Fig. 6:
It's unclear which subset of simulations were used in the plotted curves (A-C). In the supplement the plots are for several different CBV changes, so what was used in this figure?
Also, the scale on the y-axis should more closely conform to the range of data being presented (independent of what is presented in the supplement).
R: We thank the reviewer for these suggestions. We have clarified the figure and its caption to specify that the plotted curves (A-C) represent a 30% local CBV (arteries and capillaries) / 30% local SO2 (veins and capillaries) increase at the simulated activity location. To further address this, we have added an explicit text in the caption to distinguish these results from the broader range of simulations provided in the Supplement.

8. Pg. 22/23, lines 412-413:
I'm unsure about the argument that the middle cortical layer displayed a wider peak, possibly due to blood pooling from deeper cortical depths. My

understanding was that the deeper cortical layers had no corresponding change in CBV or SO2. If that's correct,

- why would the peak broaden into the deeper layers?
- Wouldn't it only spread into the more superficial layers?
- How are you quantifying the width of the peak?

R: We thank the reviewer for this insightful comment regarding the spatial distribution of the BOLD signal. Regarding the broadening into deeper layers: While the reviewer is correct that venous drainage flow is directed toward the pial surface, the broadening of the peak into deeper layers in our model is not driven by downstream blood pooling. Instead, it results from the global hemodynamic changes (the first part of our simulation) that affect the entire vascular tree, including the segments of penetrating vessels that reside below the site of local activation. Additionally, the complex 3D architecture of the capillary bed in 3D VAMOS means that even a localized change in SO2 and CBV can influence the magnetic field gradients in the immediate extravascular vicinity, including voxels slightly deeper than the primary activation site. Regarding the quantification of the peak width: We clarify that the 'width' was assessed by comparing the spatial extent of the ΔBOLD depth profiles across different simulation scenarios. This was a comparative analysis of the signal's FWHM characteristics across the depth-dependent traces.

9. Pg. 36, line 721:
Did the radii have any continuity from one vessel segment to another in the capillary network?
R: Yes, the synthetic capillary network maintains radius continuity across adjacent segments. During the generation of the 3D VAMOS network, we implemented a local "smoothing" constraint ensuring that neighboring vessel segments are assigned similar radii. This approach avoids unphysiological, abrupt changes in vessel diameter at bifurcations or along continuous paths, thereby ensuring that the morphological properties of the capillary bed remain consistent and biologically plausible. We have clarified this implementation in the Methods section (pg. 39, line 841).

10. Pg. 39, line 772:
Can you please elaborate on the purpose or motivation for including the feature to connect all V5 veins?
Are there any references that motivate including this feature?
R: The inclusion of connections between V5 veins (large intracortical veins) is motivated by the known presence of deep venous plexuses and collateral connections at the gray-white matter border. Anatomical studies of the human and mouse cerebral cortex have identified principal intracortical veins (PICVs) that serve as the primary drainage route for deep cortical layers [Stamenkovic et al., 2025; Kara et al., 2025; Havlicek, M., & Uludağ, K. (2020)]. These vessels often exhibit horizontal anastomoses or connections at deeper laminar depths, which facilitate the efficient transport of deoxygenated blood from the deep parenchyma toward the pial surface. By including these connections, 3D VAMOS more accurately represents the 'sink' effect of the venous system, which is crucial for modelling the spatial specificity of the BOLD signal and the influence of large-vessel 'blooming' artifacts across cortical depth. We have added references to these anatomical features in the Discussion section.

REFERENCES:
Stamenkovic, S., Schmid, F., Gurler, G. et al. Impaired capillary-venous drainage contributes to gliosis and demyelination in mouse white matter during aging. Nat Neurosci 28, 1868–1882 (2025). https://doi.org/10.1038/s41593-025-02023-z

Kara, Prakash, et al. Sensory-evoked Hemodynamics and the Topology of Blood Vessels in the White Matter Below the Neocortex. (2025) JOURNAL OF CEREBRAL BLOOD FLOW AND METABOLISM. Vol. 45. No. 1. 2455.
Havlicek, M., & Uludağ, K. (2020). A dynamical model of the laminar BOLD response. NeuroImage, 204, 116209. https://doi.org/10.1016/j.neuroimage.2019.116209

11. Pg. 40:
For calculating the IV signal contributions, can you please be more explicit about which quadratic expression was used from Uludag 2009?
Also, you state that the relaxation rate was weighted by the corresponding blood volume; but the blood volume also appears in Eq. (5). Is there not some risk of double-dipping the effect of CBV and totally down-weighting the IV contribution?
R: We thank the reviewer for this technical clarification. We have updated the manuscript to explicitly refer to the quadratic expression from Uludağ et al. [2009], which defines the intrinsic transverse relaxation rate of blood as a function of oxygen saturation. We have also added the definition of the arterial intrinsic relaxation rate to ensure completeness. Regarding the concern over 'double-dipping' the effect of CBV: the weighting of the relaxation rate represents a volume-weighted average of the different blood compartments (arterial and venous) to determine the effective intravascular signal contribution.

12. Pg. 43, Eqs (9) and (10):
I object to the use of the variable R2'. R2' is a relaxation rate with units of inverse time. What the authors have listed as "R2'" is the signal in the presence of R2' decay. The two are not the same.
R: We thank the reviewer for pointing out this inconsistency in our notation. We agree that R2' strictly refers to the transverse relaxation rate with units of inverse time [1/s], whereas our variable represented the MRI signal component associated with that relaxation. To avoid confusion and ensure physical accuracy, we have updated the variable throughout the manuscript to $S_{R2'}$. This notation more clearly distinguishes the signal component from the relaxation rate itself.

13. Pg. 46, line 911: Since infinite cylinders can extend across cortical layers, how was the CBV across layers controlled?
R: To control the cerebral blood volume across specific cortical layers in the RADOC model, we defined a spatially-constrained simulation domain for each layer. The in-plane dimensions of this domain were set to several hundred micrometers—a scale significantly larger than the maximal water diffusion path—to ensure that the BOLD signal contribution is statistically representative and free from boundary artifacts. Within this defined volume, cylindrical segments are iteratively populated with orientations and vessel radius drawn from a random distribution. The algorithm continues adding these segments until the target CBV for that specific laminar depth is achieved. While the cylinders are modeled as 'infinite' to avoid unphysiological end-cap effects (which could introduce artificial magnetic susceptibility gradients), their contribution to the total volume fraction is strictly calculated based on their intersection with the defined laminar boundaries (simulation domain). This ensures that the CBV is precisely controlled for each layer independently, regardless of the vessel length. We have added a detailed description of this process to the Methods section.

Minor Comments:

14. Abstract: Please state what VAMOS stands for
R: Done

15. Pg. 10, line 203:
Is there a reference for Murray's law that can be provided?
Please state what Rparent and Rdaughter1/2 are.
Why was k = 2 chosen?
R: The branching logic in our model is based on the generalized power-law relationship R^kparent = R^kdaughter1 + R^kdaughter2, where R^kparent represents the radius of the proximal stem vessel, and R^kdaughter1 and R^kdaughter2 represent the radii of the two distal branches. While Murray's Law—derived from the principle of minimum metabolic work—typically utilizes an exponent of k=3 [Murray CD, 1926], we utilized an exponent of K=2 for the 3D VAMOS framework. This choice follows the area-preservation law, which dictates that the total cross-sectional area remains constant across a bifurcation. From a physiological perspective, an exponent of K=2 is characteristic of larger vessels where it is necessary to maintain constant blood flow velocity and minimize pulse wave reflections [Kassab G. S. (2006)]. From a computational and modelling standpoint, utilizing k=2 serves as a robust simplification that prevents vessel radii from tapering too aggressively as the network depth increases. This ensures a stable and physiologically plausible CBV across the simulated volume, avoiding the 'thinning' artifacts that can occur in complex synthetic trees when k=3 is applied strictly to every microvascular junction. We have updated the Methods section to explicitly define these parameters and include the relevant citations.

REFERENCES:
Murray, C. D. (1926). The Physiological Principle of Minimum Work: I. The Vascular System and the Cost of Blood Volume. Proceedings of the National Academy of Sciences, 12(3), 207-214.
Kassab G. S. (2006). Scaling laws of vascular trees: of form and function. American journal of physiology. Heart and circulatory physiology, 290(2), H894–H903. https://doi.org/10.1152/ajpheart.00579.2005

16. Pg. 11, line 223: Please define "ROC"
R: The full definition of ROC was first introduced in the Introduction (p. 4, line 96). However, we have chosen to rename this term RADOC (RAndomly Distributed Oriented Cylinders) for improved clarity throughout the manuscript.

17. Pg. 12, Fig. 2:
Labelling the five arteries and veins in Fig. 2B as A1–A5 and V1–V5 is confusing as this is the same notation used to label the cortical penetration depths. I'd suggest using an alternate labelling scheme.
The caption for Fig. 2C is not very informative, please provide more explanation and/or annotation of the actual figure (e.g., spatial orientation)
R: We appreciate the reviewer's feedback regarding the labelling of the vascular segments. The notation A1–A5 and V1–V5 was originally chosen to maintain a direct correspondence with the five cortical penetration depths; for instance, vessels labeled A1 or V1 terminate within the superficial laminae, while those labeled A5 or V5 extend to the deepest cortical layers. However, to resolve any ambiguity between the vascular identity and the depth levels themselves, we

have updated the notation to [e.g., A_{d1}] and clarified this relationship in the text.

Furthermore, we have expanded the caption for Fig. 2C to include a more detailed explanation of the model's spatial orientation (defining the pial surface and white matter boundaries) and provided additional annotations to guide the reader through the representative vascular architectures.

18. Pg. 22, line 394: I was unable to find the following references: Bohraus et al., 2023; Guidi et al., 2016. I did not look exhaustively for other references, but please confirm the remaining references are provided.
R: References has been checked. Thank you for the comment.

19. Pg. 42, Eq. (7): On line 853, it says “response of a finite cylinder”, this should be “infinite” cylinder.
R: Done

Reviewer #2: General comments

1. According to section 4.2 (page 39), the process of spatial encoding is not considered in the model, which limits the significance of the simulations when compared to real-world data. This also suggests that the cortical profiles are idealized and lack blurring, e.g. caused by a specific point-spread function (PSF). Such a limitation may be of greater importance for the simulated SE results, since (unlike the amplitude) the envelope of the spin echo decays with R2* rather than R2 (i.e., there is also a gradient-echo contribution during the readout).
R: We thank the reviewer for this insightful observation regarding the impact of spatial encoding and the resulting point-spread function. We acknowledge that the current 3D VAMOS framework focuses on the biophysical formation of the BOLD signal within the vascular environment and does not yet simulate the specific artifacts introduced by MRI gradient sequences, such as T2* dephasing during the readout [Goense et al., 2008]. As the reviewer correctly notes, this results in 'idealized' laminar profiles that lack the spatial blurring and signal decay characteristics of real-world fMRI data. To address the significance of these acquisition effects, we recently investigated the impact of echo-train length (ETL) and k-space sampling in echo-planar imaging (EPI) readouts [van Horen et al., 2023]. This work demonstrates how the effective PSF and T2* contributions during the readout can shift and blur the signals, particularly in SE-EPI. While incorporating full spatial encoding is beyond the scope of the current structural-biophysical validation, it represents a primary direction for our future developments. We have updated the Discussion section to explicitly discuss how the PSF and sequence-specific parameters (like ETL) act as boundary conditions for the translatability of our simulated profiles to experimental 7T data.

2. I understand that VASO and ASL have not yet been included in the model though this would be of fundamental interest given the discussions regarding an improved spatial specificity of these techniques.

- At this point, it remains unclear what will be taken into account in such implementations, since specific activation-induced flow and/or volume changes are already entered as model parameters.

- Will this exclusively involve modeling the primary contrast mechanism, or will additional emphasis be placed on BOLD signal contaminations associated with these methods?
- I also assume that such integration might not be meaningful without consideration of a spatial encoding scheme?

R: We thank the reviewer for these insightful comments and agree that extending the framework to include VASO and ASL contrasts is of considerable interest.

In the current implementation, activation-induced changes in physiological parameters such as blood flow and volume are incorporated as prescribed inputs, rather than being dynamically simulated. As such, the present framework focuses on linking vascular architecture to signal formation, rather than modelling the full chain of hemodynamic processes underlying these changes.

Future extensions aimed at incorporating VASO and ASL will build upon this foundation by explicitly modelling their primary contrast mechanisms. For VASO, this will involve simulating blood volume–dependent signal changes, whereas for ASL, the focus will be on magnetically labeled inflowing blood and its exchange with tissue.

Importantly, we agree that a comprehensive implementation should not be limited to the primary contrast mechanisms alone. In particular, BOLD contamination effects are known to influence both VASO and ASL signals, and their interaction with the underlying vascular architecture can be non-negligible. Accordingly, a key objective of future work will be to integrate these competing contrast contributions within a unified modelling framework, allowing for a more realistic representation of the measured signals.

We also concur that the interpretation of such signals is inherently linked to the spatial encoding scheme. While the current framework operates at the level of signal generation, independent of acquisition-specific encoding, future developments, as mentioned in the manuscript and in the previous question, will consider the incorporation of spatial encoding effects to enable more direct comparison with experimental data. This will be particularly important for assessing the spatial specificity of different imaging modalities in the presence of realistic vascular geometries.

We clarify these points in the revised manuscript to better delineate the current scope of the model and the planned direction of future developments.

3. It appears that the model assumes a step-wise change of oxygen saturation when going from arteries to capillaries to veins (likely an acceptable simplification), whereas in a real scenario, a gradual decrease in saturation would be expected. Is there any information available regarding the quality of such an approximation?

R: We acknowledge that the 3D VAMOS framework currently utilizes a step-wise approximation for oxygen saturation SO_2 across arteries, capillaries, and veins, rather than a continuous physiological gradient. While oxygen extraction occurs gradually along the vascular tree in-vivo, this compartmentalized approach is a well-established standard in biophysical BOLD modelling [Ogawa et al., 1993]. The quality of this approximation remains high because the BOLD signal is primarily driven by the paramagnetic effects of deoxygenated hemoglobin, which is most concentrated in the venous and capillary beds. By assigning discrete, physiologically plausible values to these compartments, we can effectively isolate the influence of vascular architecture on the laminar BOLD profile without the confounding complexity of dynamic oxygen-transport modelling. This methodology ensures consistency with existing literature and experimental data derived from compartmental pO_2 measurements [Shih et al., 2012; Stamenkovic et al., 2025]. Although this simplification may slightly underestimate intra-capillary SO_2 heterogeneity, it provides a robust and validated baseline for

simulating the spatial characteristics of the BOLD response across cortical depth. This comment has been included in the Discussion section.

4. According to the second paragraph on page 44, exchange between the intra- and extravascular compartments was not integrated (“impermeable vessel walls)”. I agree that this is an acceptable simplification for studies of the BOLD response. However, since the authors mention future expansions of the framework to include, e.g. CBF measurements, this will become relevant.
R: We thank the reviewer for this important observation and agree that the assumption of impermeable vessel walls represents a simplification that may become limiting in the context of extended modelling frameworks, particularly those incorporating CBF or more detailed oxygen transport dynamics. While this assumption is appropriate for the current focus on BOLD signal formation, we recognize that exchange between intra- and extravascular compartments can play a relevant role in more comprehensive physiological models. In this regard, we are currently developing an extension of the framework that explicitly accounts for vascular exchange processes [Rundfeldt et al., 2026]. This approach aims to relax the impermeability assumption by numerically simulating the exchange of water and dissolved molecules, such as oxygen, between compartments, thereby enabling a more physiologically complete representation of tissue–blood interactions. We agree that such an extension will be essential for future applications involving coupled hemodynamic and metabolic modelling. We clarify this point in the revised manuscript.

5. It seems that some of the citations in the text are missing in the References. Examples include Davis et al., 1998; Bohraus et al., 2023; Guidi et al., 2016, Guidi et al., 2020.
R: Thank you for noticing this problem. References has been checked through the manuscript accordingly.

6. Line 81: I do not think that the abbreviation “T” of the SI unit “tesla” needs to be defined in this context.
R: Done

7. I suggest to avoid the abbreviation ROC for “randomly oriented cylinders” (defined in line 95 and used throughout the text) as it is not really necessary and might be confused with the far more common abbreviation for “receiver operating characteristic”, which is used for assessing the performance of a binary classifier model.
R: Thank you for the suggestion. The abbreviation has been changed through the manuscript to RADOC that stands for “Randomly Distributed Oriented cylinders”

8. Line 289: Remove “vessels” at the end of the sentence “… and veins (blue and purple) vessels.”
R: Done

9. Lind 856: “267.5E6” should be “267.5 × 106”.
R: Done
