# Supplementary material for: A synthetic 3D human cerebrovascular model informed by histology for simulating the cortical depth-dependent BOLD fMRI signal
Source: PLoS Comput Biol. 2026 Aug 3;22(8):e1014576. doi: 10.1371/journal.pcbi.1014576 (PMC13450845; doi:10.1371/journal.pcbi.1014576)
Supplement: Supplementary file 2 [file pcbi.1014576.s003.pdf]

Response to Reviewers (Second Round)
Reviewer #1

This is a resubmission of a previously rejected manuscript about a simulation framework referred to as "3D VAMOS" to study blood oxygenation level-dependent (BOLD) signal changes in (task-based) functional magnetic resonance imaging (fMRI). It has undergone substantial revision, largely by expanding the Discussion section are well as clarifying/justifying specific assumptions in the developed model.

As already stated in my initial evaluation, an efficient simulation tool that allows meaningful studies and can thus achieve a better understanding of relationships of the local vascular architecture and hemodynamic response is likely to be of relevant reader interest. I acknowledge that the authors have carefully and sufficiently addressed my earlier critique points. I have no further comments.

R: We sincerely thank the reviewer for their positive feedback and invaluable guidance throughout the review process. We appreciate their recognition of the study's value and are confident that their insightful suggestions have significantly improved the quality of our manuscript. Thank you!

Reviewer #2:

Báez-Yáñez et al. present a modelling framework for brain vasculature and functional MRI signals based on empirical measurements of brain vascular properties.

Unfortunately, the mentioned github repository was not publicly accessible at the time I tried it, so I can't comment on any aspects regarding the implementation and it's user-friendliness.
R: We sincerely apologize for the inconvenience caused by the temporary accessibility issue with our GitHub repository. We have verified and resolved the permissions; the repository is now fully public and functional. The source code can be accessed at the following link:
https://github.com/baezprecisionimaging-source/3dVAMOS.

Overall, the paper provides a comprehensive overview of their modelling framework and would benefit from a more thorough description of the computational aspects and a more comprehensive comparison with other approaches.
R: We would like to express our sincere gratitude to Reviewer #2 (Prof. Dr. Saskia Bollmann) for your constructive, insightful, and clear feedback. Your comments were exceptionally helpful in identifying areas for clarification and have improved the quality and readability of our manuscript. We have addressed all comments below, with corresponding changes implemented in the revised manuscript.

In detail,

1) can the authors comment on the required computational resources and run-times required for these simulations.

R: We thank the reviewer for this important question. We have updated the manuscript to include details regarding the computational requirements. The simulations were executed on a standard desktop workstation equipped with a 22-core processor and 62 GB of memory RAM. Under these hardware configurations, a single complete simulation/experiment across cortical depth for one brain region model takes approximately 6 hours to complete. We have added this information to the Discussion section under the subtitle "Computational demands and simulation runtimes" (pg. 32) of the revised manuscript:

Computational demands and simulation runtimes

The simulation runtimes were benchmarked using a standard desktop workstation configuration. The hardware environment utilized a 22-core (Intel Xeon CPU) computer paired with 62 GB of memory RAM. Under these baseline computational conditions, executing a single, complete depth-dependent BOLD simulation experiment for a single brain region required an average runtime of approximately 6 hours. This processing time scales with the structural complexity and spatial dimensions of the target 3D human vascular network, demonstrating that detailed laminar BOLD modelling can be achieved on standard local infrastructure. Nonetheless, the framework is highly scalable; leveraging high-performance computing clusters can drastically reduce overall simulation times and efficiently facilitate the parallel processing required for the statistical averaging of multiple simulation runs.

2) Further, given that a large number of empirically defined parameters enter the simulations, but the 'final' output is the depth-dependent percent signal change, a sensitivity analysis would be very insightful, assessing, for example, the impact of artery-to-vein ration, vessel radius, CBV, Grubb constant, etc.

R: We thank the reviewer for this highly insightful suggestion. We fully agree that given the multi-parameter nature of the simulation framework, a comprehensive sensitivity analysis is vital for a deeper understanding of the model's behavior. However, because our framework introduces a wide array of interconnected vascular and hemodynamic variables, a rigorous sensitivity analysis represents a massive computational and statistical undertaking. We feel that integrating it here would drastically alter the focus and scope of this foundational manuscript, which aims to validate the core simulation tool itself. To address this, we are currently developing a dedicated, follow-up study utilizing a biophysics-informed machine learning approach to systematically map the entire parameter space and learn how parameter combinations resemble empirical BOLD responses. In addition, we have currently used the 3D VAMOS model to compute VASO fMRI signals and thus characterizing the sensitivity and specificity of this sequence acquisition to CBV and SO2 changes (this has been formatted in abstract form to the ESMRMB 2026 and we have included a proof-of-concept figure below – although this will not be included in the manuscript). To ensure this limitation and future direction are clear to the reader, we have added a dedicated paragraph to the Future studies

section (pg. 34) outlining the importance of a sensitivity analysis and discussing the anticipated impact of these key parameters.

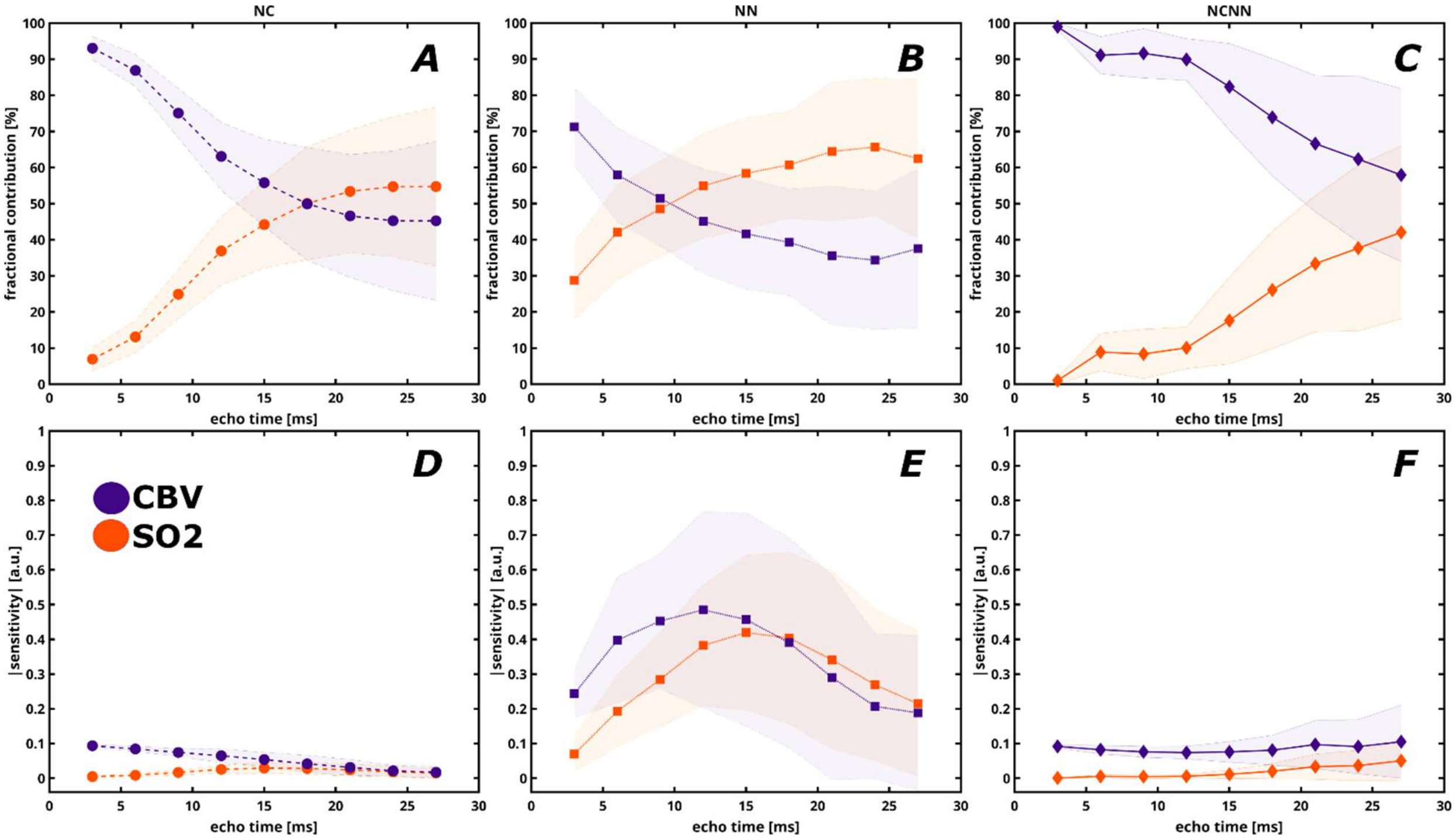

CBV and SO_2 sensitivity analysis for VASO and BOLD signal changes (Proof-of-Concept): Analysis of hemodynamic signal origins across echo times (TE). (A) Fractional contributions of SO2 and CBV to the blood-nulled signal (VASO-like). (B) Contributions to the non-nulled signal (BOLD-like). (C) Contributions to the NC/NN ratio, representing the isolated CBV-weighted contrast. Note the increase in oxygenation contribution as TE increases for both NC and NN conditions, highlighting the trade-off between physiological specificity and signal weighting in isolating CBV contributions. (D-F) Sensitivity of the signals (SNR) to changes in SO2 and CBV, respectively. The lines with dots, squares, and diamonds represent the mean values of the parameter space, while the shaded areas represent the standard deviations for the NC-, NN-, and ratio conditions, respectively.

Added paragraph to the manuscript:

A key characteristic of the 3D VAMOS framework is its reliance on a comprehensive set of empirically defined vascular and hemodynamic parameters to simulate depth-dependent percent signal changes. While this multi-parameter architecture allows for highly detailed modelling, a rigorous sensitivity analysis evaluating the isolated and joint impact of variables such as the artery-to-vein ratio, vessel radius, baseline CBV, and Grubb’s constant remains a critical next step to fully map the model’s behavior. Given the high dimensionality of this parameter space, a brute-force sensitivity analysis represents a massive computational undertaking that falls outside the foundational scope of the current validation study. To address this, ongoing work is leveraging a biophysics-informed machine learning approach to systematically map the entire

parameter space and learn how complex parameter combinations converge to resemble empirical BOLD responses. Furthermore, the utility of the framework will be extended to explore alternative contrast mechanisms, such as vascular space occupancy (VASO) fMRI signals to characterize the sensitivity and specificity of CBV and SO2 alterations. Exploring these interconnected parameters will be vital for refining the predictive accuracy of future laminar fMRI interpretations.

3) There exist a few other modelling frameworks to simulate depth-dependent BOLD signal changes (Chaussé et al., 2025; Markuerkiaga et al., 2016), but also (Hartung et al., 2025) (admittedly a pre-print) as well as work from the authors themselves (Báez-Yáñez et al., 2025). Can the authors provide more details about the commonalities and differences between these different tools, and what makes the current approach unique?

R: We thank the reviewer for highlighting these relevant modelling frameworks, as placing our framework within this broader literature is essential for clarifying its specific utility.

To address both the commonalities and distinct differences comprehensively, we have added a dedicated subsection to the revised manuscript (pg.31), which explicitly compares the vascular architecture inputs, biophysical modelling assumptions, and computational demands across these different tools.

"To date, five computational frameworks share the fundamental goal of bridging mesoscopic vascular anatomy and hemodynamic responses to predict empirical fMRI changes, whether at the voxel level or across cortical depth [Aghaeifar et al., 2025; Báez-Yáñez et al., 2025; Chaussé et al., 2025; Hartung et al., 2025; Markuerkiaga et al., 2016]. These pipelines incorporate varying MRI sequences (e.g., GE, SE, bSSFP), dimensionalities (2D vs. 3D), and field perturbation mechanics (analytical or Fourier-based magnetic field calculations). A shared objective among the laminar frameworks is accounting for how signal contributions across cortical laminae are modulated by large intracortical veins [Báez-Yáñez et al., 2025; Markuerkiaga et al., 2016]. While 3D VAMOS shares this core objective with established tools like Markuerkiaga et al. (2016) and Hartung et al. (2025), it diverges significantly in dimensionality, species origin, and pipeline customization. While earlier iterations heavily leveraged idealized or static vascular geometries, 3D VAMOS integrates fully customizable, anatomically realistic microvascular networks with dynamic, depth-dependent adjustments [Markuerkiaga et al., 2016]. Furthermore, in contrast to our previous work [Báez-Yáñez et al., 2025], 3D VAMOS introduces the simulation of synthetic, realistic human vascular models, similar to the work of Hartung et al. (2025). This structural advancement highlights the distinct uniqueness of our framework. A current limitation of the 3D VAMOS model, in comparison to the framework by Hartung et al. (2025), is the lack of time-dependent hemodynamics across the vascular network. Nevertheless, 3D VAMOS remains advantageous because it allows users to systematically isolate and manipulate intricate spatial parameters, including local artery-to-vein ratios, vessel densities, vessel-dependent oxygen

saturation, and tortuosity, within a highly accessible and flexible simulation pipeline.”

This comment has been included in the Discussion section (pg. 31)

4) It would be helpful for the reader to connect the experiments described in the methods sections with the results/figures. For example, where are the results for the different SO2 levels in veins (l886ff)?

R: Thank you for the suggestion. We have included a more specific description of the oxygen saturation results. “The SO_2 results in Figures 4 and 5 use solid lines with dots to denote the mean values across the various oxygen saturation scenarios, with shaded regions representing the standard deviation computed across several Monte Carlo simulations.” This has been included in the Methods section and the figure caption in Fig.4 and Fig.5.

5) Last, in Figure 2E, I could not see any difference between the vessels with tortuosity 1.2 and 1.5 in the zoomed-in patch. Maybe a different visualisation might be more suitable?

R: We thank the reviewer for their keen eye and for catching this error. Upon review, we realized that the incorrect sub-panel had been accidentally duplicated in the zoomed-in patch for tortuosity values 1.2 and 1.5. We apologize for this oversight. Figure 2E has been updated with the correct visualization, which now clearly shows the structural differences between the two tortuosity levels.

Journal Requirements:

1) If the reviewer comments include a recommendation to cite specific previously published works, please review and evaluate these publications to determine whether they are relevant and should be cited. There is no requirement to cite these works unless the editor has indicated otherwise.
R: Done. Suggested publications are in order and added properly.

2) Please ensure that the CRediT author contributions listed for every co-author are completed accurately and in full.
At this stage, the following Authors/Authors require contributions: Mario Gilberto Baez-Yanez, Jeroen C.W. Siero, Matthias J.P. van Osch, and Natalia Petridou. Please ensure that the full contributions of each author are acknowledged in the "Add/Edit/Remove Authors" section of our submission form.
The list of CRediT author contributions may be found here: https://journals.plos.org/ploscompbiol/s/authorship#loc-author-contributions
R: Done

3) Please upload all main figures as separate Figure files in .tif or .eps format. For more information about how to convert and format your figure files please see our guidelines: https://journals.plos.org/ploscompbiol/s/figures
R: Done

4) Please upload a copy of Figures FIG 1 A-D, 2A-H, 3A-H, 4A-D, 5A-F, and 6A-D which you refer to in your text on pages 6, 9, 13, 15, and 18.21. Or, if the figure is no longer to be included as part of the submission please remove all reference to it within the text.
R: Done

5) We have noticed that you have uploaded Supporting Information files, but you have not included a list of legends. Please add a full list of legends for your Supporting Information files after the references list.
R: Done

6) Please provide a complete Data Availability Statement in the submission form, ensuring you include all necessary access information or a reason for why you are unable to make your data freely accessible. If your research concerns only data provided within your submission, please write "All data are in the manuscript and/or supporting information files" as your Data Availability Statement.
R: Done

7) Please amend your detailed Financial Disclosure statement. This is published with the article. It must therefore be completed in full sentences and contain the exact wording you wish to be published.
R: Done

i) State the initials, alongside each funding source, of each author to receive each grant. For example: "This work was supported by the National Institutes of Health (####### to AM; ###### to CJ) and the National Science Foundation (###### to AM)."
R: Done

ii) State what role the funders took in the study. If the funders had no role in your study, please state: "The funders had no role in study design, data collection and analysis, decision to publish, or preparation of the manuscript."
R: Done

iii) If any authors received a salary from any of your funders, please state which authors and which funders..
If you did not receive any funding for this study, please simply state: u201cThe authors received no specific funding for this work.u201d
R: Done

7) We have amended your Competing Interest statement to comply with journal style. We kindly ask that you double check the statement and let us know if anything is incorrect.
R: Done
