# Supplementary material for: A synthetic 3D human cerebrovascular model informed by histology for simulating the cortical depth-dependent BOLD fMRI signal
Source: PLoS Comput Biol. 2026 Aug 3;22(8):e1014576. doi: 10.1371/journal.pcbi.1014576 (PMC13450845; doi:10.1371/journal.pcbi.1014576)
Supplement: Supplementary file 3 [file pcbi.1014576.s001.docx]

**A synthetic 3D human cerebrovascular model informed by histology for simulating the cortical depth-dependent BOLD fMRI signal**

Mario Gilberto Báez-Yáñez^1^, Jeroen C.W. Siero^1,3^, Matthias J.P. van Osch^2^ and Natalia Petridou^1^

^1^Translational Neuroimaging Group, Center for Image Sciences, University Medical Center Utrecht, Utrecht, the Netherlands

^2^C.J. Gorter MRI Center, Department of Radiology, Leiden University Medical Center, Leiden, the Netherlands

^3^Spinoza Centre for Neuroimaging Amsterdam, Amsterdam, the Netherlands.

***S1 Appendix***

In this section, we present all results related to the proxies of hemodynamic changes associated with the simulated neuronal activity.

As described in the *Results* section of the manuscript, the simulations were organized into six conditions: three corresponding to different cortical depth locations, and three corresponding to distinct vessel-specific changes in CBV and SO_2_ in response to the simulated neuronal activity. The simulated neuronal activity was centered at superficial (0.35 mm; ***panel A in S1 Appendix***), middle (1.0 mm; ***panel B in S1 Appendix***), and deep (1.5 mm; ***panel C in S1 Appendix***) cortical depths.

The vessel-specific changes simulated were as follows:

**(I) Capillaries and arteries dilated locally**, i.e., CBV changed by 0 to 30%, and SO_2_ changed locally in veins and capillaries by a similar range (0–30%) at the respective activity location.

**(II) Only capillaries dilated locally**, with CBV varying from 0–30%, and capillary SO_2_ changing from 0–30% at the respective activity location.

**(III) All vessels dilated locally**, with CBV and SO_2_ each changing from 0–30% at the respective activity location.

No permutations between CBV and SO_2_ changes were simulated.

The results are displayed according to the corresponding condition number for all magnitudes of change.

**(I) Capillaries and arteries dilated locally**, i.e., CBV changed by 0 to 30%, and SO_2_ changed locally in veins and capillaries by 0 to 30% at the respective activity location.

**Fig A** Capillaries and arteries dilated locally, ΔCBV = 0%; ΔSO_2_ = 0%. The simulated neuronal activity was centered at superficial (0.35 mm; ***panel A***), middle (1.0 mm; ***panel B***), and deep (1.5 mm; ***panel C***) cortical depths.

**Fig B** Capillaries and arteries dilated locally, ΔCBV = 10%; ΔSO_2_ = 10%. The simulated neuronal activity was centered at superficial (0.35 mm; ***panel A***), middle (1.0 mm; ***panel B***), and deep (1.5 mm; ***panel C***) cortical depths.

**Fig C** Capillaries and arteries dilated locally, ΔCBV = 20%; ΔSO_2_ = 20%. The simulated neuronal activity was centered at superficial (0.35 mm; ***panel A***), middle (1.0 mm; ***panel B***), and deep (1.5 mm; ***panel C***) cortical depths.

**Fig D** Capillaries and arteries dilated locally, ΔCBV = 30%; ΔSO_2_ = 30% **(as in *Fig 6 in the manuscript*).** The simulated neuronal activity was centered at superficial (0.35 mm; ***panel A***), middle (1.0 mm; ***panel B***), and deep (1.5 mm; ***panel C***) cortical depths.

**(II) Only capillaries dilated locally**, with CBV varying from 0–30%, and capillary SO_2_ changing from 0–30% at the respective activity location.

**Fig E** Only capillaries dilated locally, ΔCBV = 0%; ΔSO_2_ = 0%. The simulated neuronal activity was centered at superficial (0.35 mm; ***panel A***), middle (1.0 mm; ***panel B***), and deep (1.5 mm; ***panel C***) cortical depths.

**Fig F** Only capillaries dilated locally, ΔCBV = 10%; ΔSO_2_ = 10%. The simulated neuronal activity was centered at superficial (0.35 mm; ***panel A***), middle (1.0 mm; ***panel B***), and deep (1.5 mm; ***panel C***) cortical depths.

**Fig G** Only capillaries dilated locally, ΔCBV = 20%; ΔSO_2_ = 20%. The simulated neuronal activity was centered at superficial (0.35 mm; ***panel A***), middle (1.0 mm; ***panel B***), and deep (1.5 mm; ***panel C***) cortical depths.

**Fig H** Only capillaries dilated locally, ΔCBV = 30%; ΔSO_2_ = 30%. The simulated neuronal activity was centered at superficial (0.35 mm; ***panel A***), middle (1.0 mm; ***panel B***), and deep (1.5 mm; ***panel C***) cortical depths.

**(III) All vessels dilated locally**, with CBV and SO_2_ each changing from 0–30% at the respective activity location.

**Fig I** All vessels dilated locally, ΔCBV = 0%; ΔSO_2_ = 0%. The simulated neuronal activity was centered at superficial (0.35 mm; ***panel A***), middle (1.0 mm; ***panel B***), and deep (1.5 mm; ***panel C***) cortical depths.

**Fig J** All vessels dilated locally, ΔCBV = 10%; ΔSO_2_ = 10%. The simulated neuronal activity was centered at superficial (0.35 mm; ***panel A***), middle (1.0 mm; ***panel B***), and deep (1.5 mm; ***panel C***) cortical depths.

**Fig K** All vessels dilated locally, ΔCBV = 20%; ΔSO_2_ = 20%. The simulated neuronal activity was centered at superficial (0.35 mm; ***panel A***), middle (1.0 mm; ***panel B***), and deep (1.5 mm; ***panel C***) cortical depths.

**Fig L** All vessels dilated locally, ΔCBV = 30%; ΔSO_2_ = 30%. The simulated neuronal activity was centered at superficial (0.35 mm; ***panel A***), middle (1.0 mm; ***panel B***), and deep (1.5 mm; ***panel C***) cortical depths.
