# Supplementary material for: A synthetic 3D human cerebrovascular model informed by histology for simulating the cortical depth-dependent BOLD fMRI signal
Source: PLoS Comput Biol. 2026 Aug 3;22(8):e1014576. doi: 10.1371/journal.pcbi.1014576 (PMC13450845; doi:10.1371/journal.pcbi.1014576)
Supplement: Peer review history

# Author comment: Author response to Decision Letter 0

**DOI:** 10.1371/journal.pcbi.1014576.r001

15 May 2026

Attachment Submitted filename: ResponsesToReviewers.pdf

# Peer review: Decision Letter 0

**DOI:** 10.1371/journal.pcbi.1014576.r002

**Authors:** Daniele Marinazzo, Jian Liu

29 Jun 2026

PCOMPBIOL-D-26-01166

A synthetic 3D human cerebrovascular model informed by histology for simulating the cortical depth-dependent BOLD fMRI signal

PLOS Computational Biology

Dear Dr. Baez-Yanez,

Thank you for submitting your manuscript to PLOS Computational Biology. After careful consideration, we feel that it has merit but does not fully meet PLOS Computational Biology's publication criteria as it currently stands. Therefore, we invite you to submit a revised version of the manuscript that addresses the points raised during the review process.

Please submit your revised manuscript by Jul 29 2026 11:59PM. If you will need more time than this to complete your revisions, please reply to this message or contact the journal office at ploscompbiol@plos.org. When you're ready to submit your revision, log on to https://www.editorialmanager.com/pcompbiol/ and select the 'Submissions Needing Revision' folder to locate your manuscript file.

Please include the following items when submitting your revised manuscript:

* A letter that responds to each point raised by the editor and reviewer(s). You should upload this letter as a separate file labeled 'Response to Reviewers'. This file does not need to include responses to formatting updates and technical items listed in the 'Journal Requirements' section below.

* A marked-up copy of your manuscript that highlights changes made to the original version. You should upload this as a separate file labeled 'Revised Manuscript with Track Changes'.

* An unmarked version of your revised paper without tracked changes. You should upload this as a separate file labeled 'Manuscript'.

If you would like to make changes to your financial disclosure, competing interests statement, or data availability statement, please make these updates within the submission form at the time of resubmission. Guidelines for resubmitting your figure files are available below the reviewer comments at the end of this letter.

As the corresponding author, your ORCID iD is verified in the submission system and will appear in the published article. PLOS supports the use of ORCID, and we encourage all coauthors to register for an ORCID iD and use it as well. Please encourage your coauthors to verify their ORCID iD within the submission system before final acceptance, as unverified ORCID iDs will not appear in the published article. Only the individual author can complete the verification step; PLOS staff cannot verify ORCID iDs on behalf of authors.

We look forward to receiving your revised manuscript.

Kind regards,

Jian Liu

Academic Editor

PLOS Computational Biology

Daniele Marinazzo

Section Editor

PLOS Computational Biology

**Additional Editor Comments (if provided):**

The manuscript demonstrates clear merit. However, certain methodological details require additional clarification to improve overall readability. Please revise the text as suggested.

**Journal Requirements:**

If the reviewer comments include a recommendation to cite specific previously published works, please review and evaluate these publications to determine whether they are relevant and should be cited. There is no requirement to cite these works unless the editor has indicated otherwise.

1) Please ensure that the CRediT author contributions listed for every co-author are completed accurately and in full.

At this stage, the following Authors/Authors require contributions: Mario Gilberto Baez-Yanez, Jeroen C.W. Siero, Matthias J.P. van Osch, and Natalia Petridou. Please ensure that the full contributions of each author are acknowledged in the "Add/Edit/Remove Authors" section of our submission form.

The list of CRediT author contributions may be found here: https://journals.plos.org/ploscompbiol/s/authorship#loc-author-contributions

2) Please upload all main figures as separate Figure files in .tif or .eps format. For more information about how to convert and format your figure files please see our guidelines:

https://journals.plos.org/ploscompbiol/s/figures

3) Please upload a copy of Figures FIG 1 A-D, 2A-H, 3A-H, 4A-D, 5A-F, and 6A-D which you refer to in your text on pages 6, 9, 13, 15, and 18.21. Or, if the figure is no longer to be included as part of the submission please remove all reference to it within the text.

4) We have noticed that you have uploaded Supporting Information files, but you have not included a list of legends. Please add a full list of legends for your Supporting Information files after the references list.

5) Please provide a complete Data Availability Statement in the submission form, ensuring you include all necessary access information or a reason for why you are unable to make your data freely accessible. If your research concerns only data provided within your submission, please write "All data are in the manuscript and/or supporting information files" as your Data Availability Statement.

6) Please amend your detailed Financial Disclosure statement. This is published with the article. It must therefore be completed in full sentences and contain the exact wording you wish to be published.

i) State the initials, alongside each funding source, of each author to receive each grant. For example: "This work was supported by the National Institutes of Health (####### to AM; ###### to CJ) and the National Science Foundation (###### to AM)."

ii) State what role the funders took in the study. If the funders had no role in your study, please state: "The funders had no role in study design, data collection and analysis, decision to publish, or preparation of the manuscript."

iii) If any authors received a salary from any of your funders, please state which authors and which funders..

If you did not receive any funding for this study, please simply state: u201cThe authors received no specific funding for this work.u201d

7)We have amended your Competing Interest statement to comply with journal style. We kindly ask that you double check the statement and let us know if anything is incorrect.

**Reviewers' comments:**

Reviewer's Responses to Questions

**Comments to the Authors:**

**Please note here if the review is uploaded as an attachment.**

Reviewer #1: This is a resubmission of a previously rejected manuscript about a simulation framework referred to as “3D VAMOS” to study blood oxygenation level-dependent (BOLD) signal changes in (task-based) functional magnetic resonance imaging (fMRI). It has undergone substantial revision, largely by expanding the Discussion section are well as clarifying/justifying specific assumptions in the developed model.

As already stated in my initial evaluation, an efficient simulation tool that allows meaningful studies and can thus achieve a better understanding of relationships of the local vascular architecture and hemodynamic response is likely to be of relevant reader interest. I acknowledge that the authors have carefully and sufficiently addressed my earlier critique points. I have no further comments.

Reviewer #2: Báez-Yáñez et al. present a modelling framework for brain vasculature and functional MRI signals based on empirical measurements of brain vascular properties.

Unfortunately, the mentioned github repository was not publicly accessible at the time I tried it, so I can’t comment on any aspects regarding the implementation and it’s user-friendliness. Overall, the paper provides a comprehensive overview of their modelling framework and would benefit from a more thorough description of the computational aspects and a more comprehensive comparison with other approaches.

In detail, can the authors comment on the required computational resources and run-times required for these simulations.

Further, given that a large number of empirically defined parameters enter the simulations, but the ‘final’ output is the depth-dependent percent signal change, a sensitivity analysis would be very insightful, assessing, for example, the impact of artery-to-vein ration, vessel radius, CBV, Grubb constant, etc.

There exist a few other modelling frameworks to simulate depth-dependent BOLD signal changes (Chaussé et al., 2025; Markuerkiaga et al., 2016), but also (Hartung et al., 2025) (admittedly a pre-print) as well as work from the authors themselves (Báez-Yáñez et al., 2025). Can the authors provide more details about the commonalities and differences between these different tools, and what makes the current approach unique.

It would be helpful for the reader to connect the experiments described in the methods sections with the results/figures. For example, where are the results for the different SO2 levels in veins (l886ff)?

Last, in Figure 2E, I could not see any difference between the vessels with tortuosity 1.2 and 1.5 in the zoomed-in patch. Maybe a different visualisation might be more suitable?

Báez-Yáñez, M.G., Siero, J.C.W., Curcic, V., van Osch, M.J.P., Petridou, N., 2025. Impact of vascular architecture, oxygen saturation, and hematocrit on human cortical depth-dependent GE- and SE-BOLD fMRI signals: A simulation approach using realistic 3D vascular networks. Imaging Neurosci. 3, imag_a_00573. https://doi.org/10.1162/imag_a_00573

Chaussé, J., Berman, A.J.L., Chen, J.J., 2025. BOLDsωimsuite: A new software suite for forward modeling of the BOLD fMRI signal. Imaging Neurosci. 3, imag_a_00519. https://doi.org/10.1162/imag_a_00519

Hartung, G., Berman, A.J.L., Sakadžić, S., Linninger, A., Boas, D.A., Polimeni, J.R., 2025. Biophysical simulations of fMRI responses using realistic microvascular models: insights into distinct hemodynamics in humans and mice. https://doi.org/10.1101/2025.10.08.680976

Markuerkiaga, I., Barth, M., Norris, D.G., 2016. A cortical vascular model for examining the specificity of the laminar BOLD signal. Neuroimage 132, 491–8. https://doi.org/10.1016/j.neuroimage.2016.02.073

**********

**Have the authors made all data and (if applicable) computational code underlying the findings in their manuscript fully available?**

The PLOS Data policy requires authors to make all data and code underlying the findings described in their manuscript fully available without restriction, with rare exception (please refer to the Data Availability Statement in the manuscript PDF file). The data and code should be provided as part of the manuscript or its supporting information, or deposited to a public repository. For example, in addition to summary statistics, the data points behind means, medians and variance measures should be available. If there are restrictions on publicly sharing data or code —e.g. participant privacy or use of data from a third party—those must be specified.

Reviewer #1: Yes

Reviewer #2: **No:**  The mentioned github repository was not accessible at the time I tried.

**********

PLOS authors have the option to publish the peer review history of their article (what does this mean?). If published, this will include your full peer review and any attached files.

If you choose “no”, your identity will remain anonymous but your review may still be made public.

**Do you want your identity to be public for this peer review?**  For information about this choice, including consent withdrawal, please see our Privacy Policy.

Reviewer #1: No

Reviewer #2: **Yes:**  Saskia Bollmann

[NOTE: If reviewer comments were submitted as an attachment file, they will be attached to this email and accessible via the submission site. Please log into your account, locate the manuscript record, and check for the action link "View Attachments". If this link does not appear, there are no attachment files.]

**Figure resubmission:**

While revising your submission, we strongly recommend that you use PLOS’s NAAS tool (https://ngplosjournals.pagemajik.ai/artanalysis) to test your figure files. NAAS can convert your figure files to the TIFF file type and meet basic requirements (such as print size, resolution), or provide you with a report on issues that do not meet our requirements and that NAAS cannot fix.

After uploading your figures to PLOS’s NAAS tool - https://ngplosjournals.pagemajik.ai/artanalysis, NAAS will process the files provided and display the results in the "Uploaded Files" section of the page as the processing is complete. If the uploaded figures meet our requirements (or NAAS is able to fix the files to meet our requirements), the figure will be marked as "fixed" above. If NAAS is unable to fix the files, a red "failed" label will appear above. When NAAS has confirmed that the figure files meet our requirements, please download the file via the download option, and include these NAAS processed figure files when submitting your revised manuscript.

**Reproducibility:**

To enhance the reproducibility of your results, we recommend that authors of applicable studies deposit laboratory protocols in protocols.io, where a protocol can be assigned its own identifier (DOI) such that it can be cited independently in the future. Additionally, PLOS ONE offers an option to publish peer-reviewed clinical study protocols. Read more information on sharing protocols at https://plos.org/protocols?utm_medium=editorial-email&utm_source=authorletters&utm_campaign=protocols

# Author comment: Author response to Decision Letter 1

**DOI:** 10.1371/journal.pcbi.1014576.r003

6 Jul 2026

Attachment Submitted filename: ResponsesToReviewers_2ndRound.pdf

# Editor report: Decision Letter 1

**DOI:** 10.1371/journal.pcbi.1014576.r004

**Authors:** Daniele Marinazzo, Jian Liu

14 Jul 2026

Dear Dr. Baez-Yanez,

We are pleased to inform you that your manuscript 'A synthetic 3D human cerebrovascular model informed by histology for simulating the cortical depth-dependent BOLD fMRI signal' has been provisionally accepted for publication in PLOS Computational Biology.

Before your manuscript can be formally accepted you will need to complete some formatting changes, which you will receive in a follow up email. A member of our team will be in touch with a set of requests.

Please note that your manuscript will not be scheduled for publication until you have made the required changes, so a swift response is appreciated.

IMPORTANT: The editorial review process is now complete. PLOS will only permit corrections to spelling, formatting or significant scientific errors from this point onwards. Requests for major changes, or any which affect the scientific understanding of your work, will cause delays to the publication date of your manuscript.

Should you, your institution's press office or the journal office choose to press release your paper, you will automatically be opted out of early publication. We ask that you notify us now if you or your institution is planning to press release the article. All press must be co-ordinated with PLOS.

Thank you again for supporting Open Access publishing; we are looking forward to publishing your work in PLOS Computational Biology.

Best regards,

Jian Liu

Academic Editor

PLOS Computational Biology

Daniele Marinazzo

Section Editor

PLOS Computational Biology

***********************************************************

The authors have revised the manuscript thoroughly and addressed all reviewer concerns. It is now ready for publication.

# Editor report: Acceptance letter

**DOI:** 10.1371/journal.pcbi.1014576.r005

**Authors:** Daniele Marinazzo, Jian Liu

PCOMPBIOL-D-26-01166R1

A synthetic 3D human cerebrovascular model informed by histology for simulating the cortical depth-dependent BOLD fMRI signal

Dear Dr Baez-Yanez,

I am pleased to inform you that your manuscript has been formally accepted for publication in PLOS Computational Biology. Your manuscript is now with our production department and you will be notified of the publication date in due course.

The corresponding author will soon be receiving a typeset proof for review, to ensure errors have not been introduced during production. Please review the PDF proof of your manuscript carefully, as this is the last chance to correct any errors. Please note that major changes, or those which affect the scientific understanding of the work, will likely cause delays to the publication date of your manuscript.

Soon after your final files are uploaded, unless you have opted out, the early version of your manuscript will be published online. The date of the early version will be your article's publication date. The final article will be published to the same URL, and all versions of the paper will be accessible to readers.

For Research, Software, and Methods articles, you will receive an invoice from PLOS for your publication fee after your manuscript has reached the completed accept phase. If you receive an email requesting payment before acceptance or for any other service, this may be a phishing scheme. Learn how to identify phishing emails and protect your accounts at https://explore.plos.org/phishing.

Thank you again for supporting PLOS Computational Biology and open-access publishing. We are looking forward to publishing your work!

With kind regards,

Anitha Samidurai

PLOS Computational Biology | Carlyle House, Carlyle Road, Cambridge CB4 3DN | United Kingdom ploscompbiol@plos.org | Phone +44 (0) 1223-442824 | ploscompbiol.org | @PLOSCompBiol
